# Investigating the Interactions of Peptide Nucleic Acids with Multicomponent Peptide Hydrogels for the Advancement of Healthcare Technologies

**DOI:** 10.3390/gels11050367

**Published:** 2025-05-17

**Authors:** Sabrina Giordano, Monica Terracciano, Enrico Gallo, Carlo Diaferia, Andrea Patrizia Falanga, Antonella Accardo, Monica Franzese, Marco Salvatore, Gennaro Piccialli, Nicola Borbone, Giorgia Oliviero

**Affiliations:** 1 IRCCS SYNLAB SDN, Via G. Ferraris 144, 80143 Naples, Italy; sabrina.giordano@synlab.it (S.G.); enrico.gallo@synlab.it (E.G.); monica.franzese@synlab.it (M.F.); marco.salvatore@synlab.it (M.S.); 2 Department of Pharmacy, University of Naples Federico II, Via D. Montesano 49, 80131 Naples, Italy; carlo.diaferia@unina.it (C.D.); andreapatrizia.falanga@unina.it (A.P.F.); antonella.accardo@unina.it (A.A.); picciall@unina.it (G.P.); nicola.borbone@unina.it (N.B.); 3 Department of Molecular Medicine and Medical Biotechnologies, University of Naples Federico II, Via S. Pansini 5, 80131 Naples, Italy

**Keywords:** peptide nucleic acids, hydrogels, chemical functionalization, multifunctional systems, healthcare applications

## Abstract

This study reports the development of peptide-based hydrogels for the encapsulation and controlled release of peptide nucleic acids in drug delivery applications. Ultrashort aromatic peptides, such as Fmoc-FF, self-assemble into biocompatible hydrogels with nanostructured architectures. The functionalization of tripeptides (Fmoc-FFK and Fmoc-FFC) with lysine (K) or cysteine (C) enables electrostatic or covalent interactions with model PNAs engineered with glutamic acid or cysteine residues, respectively. Hydrogels were polymerized in situ in the presence of PNAs, and component ratios were systematically varied to optimize mechanical properties, loading efficiency, and release kinetics. The formulations obtained with a 1/10 ratio of Fmoc-FF(K or C)/Fmoc-FF provided an optimal balance between structural integrity and delivery performance. All hydrogel formulations demonstrated high stiffness (G′ > 19,000 Pa), excellent water retention, and minimal swelling under physiological conditions (ΔW < 4%). The release studies over 10 days showed that electrostatic loading enabled faster and higher release (up to 90%), while covalent bonding resulted in slower, sustained delivery (~15%). These findings highlight the tunability of the hydrogel system for diverse therapeutic applications.

## 1. Introduction

In the rapidly evolving field of nanomedicine, the development of advanced materials capable of performing multifunctional tasks within biological systems is crucial for innovative healthcare applications [1]. Among these materials, peptide-based hydrogels have emerged as promising nanostructured candidates due to their unique combination of biocompatibility, biodegradability, and tunable physicochemical properties [2,3]. These materials mimic biological molecules, making them suitable for a wide range of applications, from tissue engineering to drug delivery, as well as biosensing applications [4,5,6].

Specifically, Fmoc-FF (*N*_α_-fluorenyl methoxycarbonyl-diphenylalanine) is one of the most studied low molecular-weight hydrogelators, providing a foundation for the design of new peptide-based gelators with improved characteristics [7,8,9].

Although the Fmoc-protecting group may exhibit some degree of in vitro toxicity, it is one of the most extensively studied systems due to its favorable self-assembly properties and is widely considered a reference for the development of peptide-based hydrogels. In particular, Fmoc-FF has been reported to exhibit very low toxicity (<5%) when combined with other peptides [10,11]. In our previous studies, we explored the potential of the Fmoc-FF hydrogelator to form mixed hydrogels in combination with tripeptides sharing the same aromatic core and containing an additional residue of Cys, Ser, Thr, or Lys at their *C*-terminus. Peptide-based mixed hydrogels have been shown to enhance reproducibility and scalability, making them more suitable for biotechnological and biomedical applications [12,13]. By incorporating different quantities of the reactive functionalities, it was possible to easily obtain materials with the desired level of reactive groups on their surface, thus enhancing their interaction with bioactive molecules, such as peptide nucleic acids (PNAs), for biomedical applications.

PNAs are synthetic analogs to natural nucleic acids characterized by a backbone of *N*–(2-aminoethyl)-glycine units linked via amide bonds. This structure lacks the negatively charged phosphate groups, resulting in reduced electrostatic repulsion and significantly enhanced binding affinity to complementary single-stranded DNA or RNA [14]. As a result, PNAs have attracted substantial relevance in the biomedical field, acting as therapeutics exploiting the antigene [15,16], antisense [17,18,19], and anti-miRNA [20,21,22] approaches, as well as serving as biosensing probes in diagnostic applications [23,24].

PNAs’ enhanced binding affinity is combined with low toxicity and excellent chemical and enzymatic stability [25]. Despite these notable advantages, PNAs’ limited cellular permeability and water solubility present challenges for medical applications. The development of effective delivery systems is therefore essential to address these limitations, ensuring enhanced bioavailability and therapeutic efficacy [26,27]. In this context, peptide-based hydrogels offer a compelling solution by providing a biocompatible and biodegradable scaffold that can stabilize PNA molecules and facilitate their controlled release at the target site.

In this study, we investigated peptide-based hydrogels as scaffolds for encapsulating PNAs for drug delivery applications. By directly polymerizing the hydrogels in the presence of PNAs, we systematically varied the concentrations and ratios of the hydrogel components to optimize their properties, including encapsulation efficiency, structural stability, and controlled release profiles. This approach aims to establish hydrogels as effective and biocompatible delivery systems, highlighting their capacity to stabilize PNAs and preserve their bioactivity in complex biological environments.

**Figure 1:**
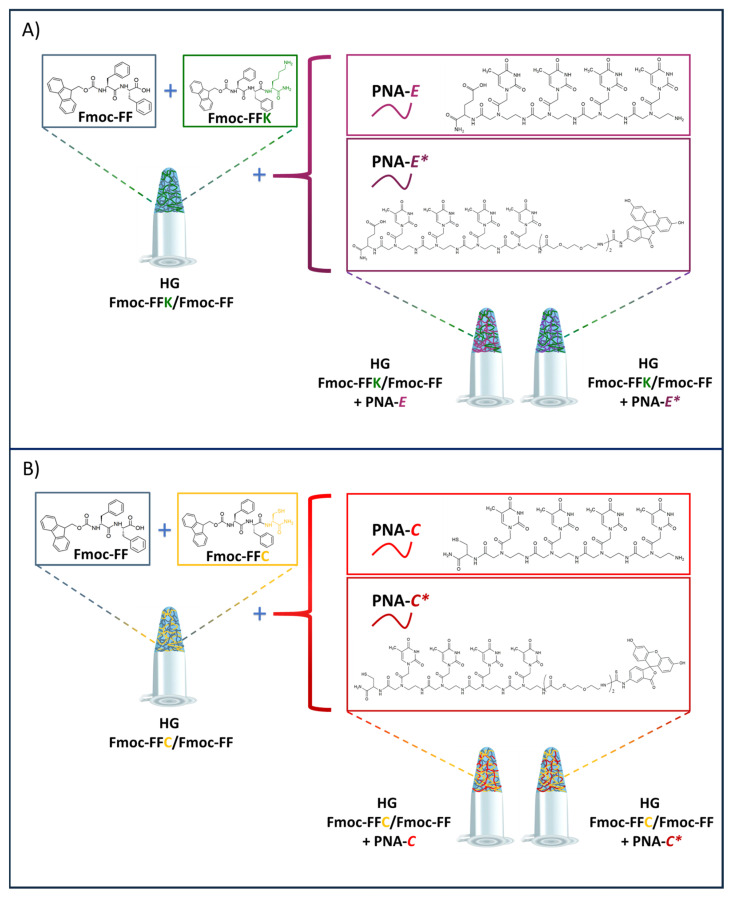
(**A**) Chemical structure and schematic representation of Fmoc-FF (N_α_-fluorenylmethoxycarbonyl-diphenylalanine), Fmoc-FFK (Fmoc-diphenylalanine-lysine), and the corresponding hydrogels (HG Fmoc-FFK/Fmoc-FF) formed through interaction with PNA sequences containing glutamic acid (E) without or with FITC labeling, referred to as PNA-E and PNA-E*, respectively. (**B**) Chemical structure and schematic representation of Fmoc-FF, Fmoc-FFC (Fmoc-diphenylalanine-cysteine), and the corresponding hydrogels (HG Fmoc-FFC/Fmoc-FF) formed through interactions with PNA sequences containing cysteine (C) without or with FITC labeling, referred to as PNA-C and PNA-C*, respectively.

We employed model PNA sequences four bases long, synthesized with and without fluorescein 5-isothiocyanate (FITC) as a fluorescent marker. Each sequence was functionalized with either a cysteine (C) [17] or a glutamic acid (E) residue at the C-terminus, facilitating covalent or electrostatic interactions with tripeptides containing cysteine (C) or lysine (K) residues within the hydrogel matrix, respectively. This design enhanced the specificity and strength of the interactions between PNAs and the hydrogel scaffold (Figure 1). The hydrogels were prepared using a DMSO/H_2_O solvent-switch approach, maintaining a 10/90 DMSO/H_2_O ratio. During the gelation process, the system transitioned from an opaque solution to a clear gel, as typically observed for Fmoc-FF hydrogels. This transformation highlights the ability of these systems to self-assemble into stable, nanostructured networks in aqueous environments. The resulting hydrogels were subjected to comprehensive characterization using advanced analytical techniques, including High-Performance Liquid Chromatography (HPLC), Electrospray Ionization Mass Spectrometry (ESI-MS), Circular Dichroism (CD), rheology, Fourier Transform Infrared (FT-IR) spectroscopy, Scanning Electron Microscopy (SEM), Proton Nuclear Magnetic Resonance (¹H-NMR), as well as optical and fluorescence microscopies.

## 2. Results and Discussion

### 2.1. Design and Synthesis of PNA Sequences

The PNA sequences synthesized for this study (Figure 1, Table 1, Figures S1–S4) were designed to be four bases long, to facilitate a chemical investigation focused on the interactions between PNA probes and Fmoc-FF mixed hydrogels in the fabrication of three-dimensional scaffolds with potential biomedical applications. Fmoc-T-OH molecules were selected as monomers because pyrimidines are more suitable than purines for manual solid-phase synthesis due to their lower steric hindrance. Additionally, thymine is the only nitrogenous base that does not require protective groups, as it lacks reactive groups on its heterocyclic ring. The PNA sequences were also synthesized by incorporating a cysteine (C) or glutamic acid (E) residue as the first monomer at the *C*-terminus of the main chain. This design was intended to promote specific interactions between the PNA probes and the tripeptides within the hydrogels. The cysteine (C) residue was included to facilitate the formation of covalent bonds with the cysteine residue of the tripeptide, particularly through disulfide linkages. The introduction of glutamic acid (E) aimed to promote electrostatic interactions due to its negatively charged side chain, which can interact with positively charged lysine (K) residues in the tripeptides. These targeted interactions were aimed at enhancing the stability and integration of the PNA probes within the peptide-based hydrogel scaffolds. The PNA sequences were also synthesized with fluorescein 5-isothiocyanate (FITC) labeling at the *N*-terminus to facilitate a better understanding of hydrogel formation. This labeling is instrumental for observing the interactions between the PNA probes and the peptide hydrogels, offering valuable insights into the dynamics of hydrogel assembly. The high absorptivity and fluorescence quantum yield of FITC enable sensitive detection of the PNA sequences during optical measurements. To improve water solubility, two units of the PEG_2_ spacer were also inserted between the FITC and the tetra-thymine framework.

### 2.2. Design and Synthesis of Peptide Building Blocks

To ensure high chemical homology, the tripeptide design should be ensured by a minor disruption of the previously identified non-covalent contact pathways responsible for both aggregation and gelation, as demonstrated in our previous studies [12,13]. Among the twenty natural amino acids, cysteine (C) and lysine (K) were selected to promote interactions with the synthesized PNAs. These functional entities can be efficiently employed for post-aggregation functionalization, facilitating the creation of responsive systems tailored for biomedical applications, including biosensing and drug delivery [28]. The two tripeptides (Fmoc-FFC and Fmoc-FFK) (Figure 1, Table 2, and Figure S5) were synthesized and purified using conventional solid-phase peptide synthesis (SPPS) standard protocols and RP-HPLC chromatography. ESI mass spectrometry, ^1^H-NMR spectroscopy, and analytical HPLC characterization were then used to confirm their identity [12,13]. These tripeptides retain the Fmoc-FF motif but also have a residue at the *C*-terminal end that provides additional properties to the resulting hydrogel that allow it to be functionalized with biologically active compounds.

### 2.3. Formulation of Multicomponent Hydrogels

**Figure 2:**
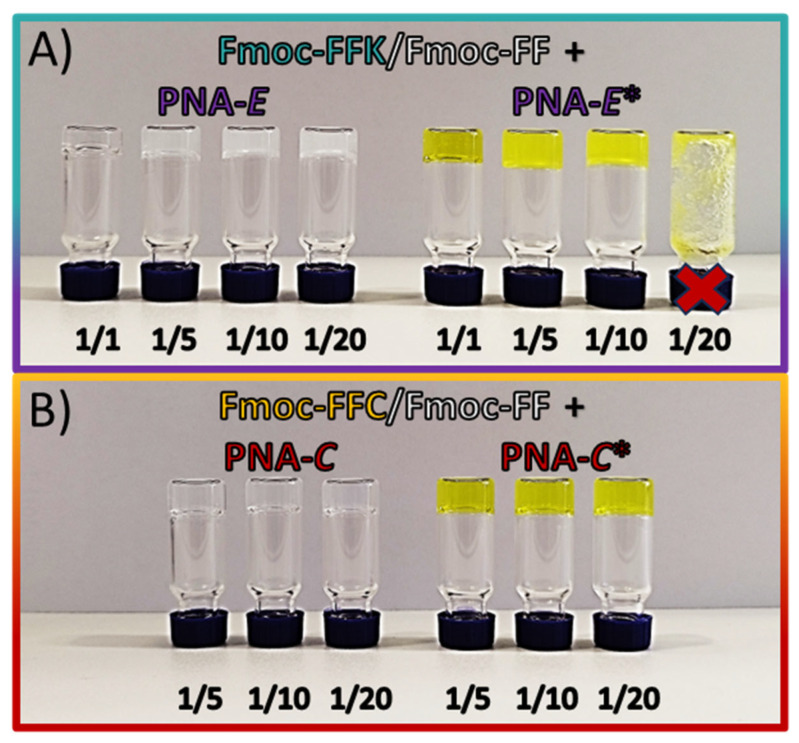
Inverted test tube for 400 µL of mixed hydrogel formulations (1.0 wt%) of (**A**) Fmoc-FFK/Fmoc-FF HGs at different ratios (1/1, 1/5, 1/10, 1/20, *w*/*w*) with 0.15 mM PNA-E (left) and 0.15 mM PNA-E* (right) solutions. (**B**) Fmoc-FFC/Fmoc-FF HGs at different ratios (1/5, 1/10, 1/20, *w*/*w*) with 0.15 mM PNA-C (left) and 0.15 mM PNA-C* (right) solutions.

To generate hydrogels, the synthesized tripeptides were combined with the Fmoc-FF and PNA molecules. The Fmoc-FFX sequences were mixed with Fmoc-FF at different ratios (1/1, 1/5, 1/10, and 1/20 *w*/*w* for Fmoc-FFX/Fmoc-FF, where X = C or K), as previously evaluated for mixed matrices [12,13]. To achieve hydrogels with varying degrees of functionalization, the tripeptide content within the mixed matrices was intentionally reduced in a stepwise manner. Subsequently, hybrid hydrogels were produced by using the solvent-switch approach while maintaining a constant DMSO/H_2_O ratio (10/90, ϕ_solvent_ = 0.10%) [29]. The addition of water caused the metastable solution to switch from opaque to clear, which is in agreement with the expected behavior of Fmoc-FF-based hydrogels [30]. To promote the incorporation of the PNA molecules into the mixed hydrogels, the solvent-switch method was slightly modified. The PNA molecules were dissolved in doubly filtered Milli-Q water, and these solutions of known concentration were then used to formulate the mixed hydrogels by the solvent-switch method. The gel formation was confirmed by the inverted test tube (Figure 2 and Figure S5). Due to the increase in opacity associated with the gel formation process, the absorbance spectra of the samples at 600 nm (long-range) were measured over time at set intervals to quantitatively determine the kinetics of gel formation. In this way, it was possible to identify the gelation times (G_t_) corresponding to the inflection point of the curves (Figure S6), as all samples show a decrease in optical density (OD) during the transition from opaque to clear. Previous studies on gelation kinetics revealed that the formation of a pure Fmoc-FFK hydrogel requires a significantly longer time (45 min) [13] compared to the pure Fmoc-FF hydrogel (2 min) [10].

Moreover, the incorporation of Fmoc-FFX tripeptides further increases the gelation time. However, the amount of tripeptide relative to Fmoc-FF does not appear to be the sole factor influencing the gelling kinetics, as shown by the gelation times listed in Table 3. The incorporation of PNA molecules generally leads to extended formation times for all samples, with the notable exception of the Fmoc-FFK/Fmoc-FF 1/20 + PNA-E system, where the gelation time is markedly reduced. This observation suggests that factors such as heteroatoms, steric hindrance, and the presence of PNA may play a significant role in influencing gel formation. Specifically, steric hindrance is particularly pronounced in the Fmoc-FFK/Fmoc-FF 1/20 + PNA-E* sample, which exhibits no gelation (Figure 2A), emphasizing how this factor can substantially hinder the gelation process.

### 2.4. Rheological Characterization

The obtained systems showed self-supporting properties, suggesting the formation of non-Newtonian behavior [31]. The resulting materials were evaluated in their mechanical response via rotational rheological analysis, reporting the viscoelastic profile (Figure S7) for each Fmoc-FFX-containing PNA [50 µM] in terms of *G*′ (storage modulus) and *G*″ (loss modulus). The analysis was conducted by performing a dynamic oscillation strain sweep (at a frequency of 1.0 Hz), extrapolating moduli values at ω = 0.1% (Table 4). The loss and storage moduli were calculated in duplicate, and no significant differences were observed.

The *G*′ > *G*″ relationship, as the consequent tan δ (*G*″/*G*′) < 1, analytically demonstrates the gel state of all the tested matrices, with a preference for elastic dissipation of the applied torque force. The linear viscoelastic region (LVE region) for both Fmoc-FFK- and Fmoc-FFC-containing matrices was found in the 0.01–8.0% stain range, in line with empty peptide-based hydrogels [12,13]. An increase of *G*′ with the amount of Fmoc-FF component was observed only for Fmoc-FFC-containing matrices. Fmoc-FFC-based HGs are more rigid at higher percentages of Fmoc-FF and less rigid at lower percentages of Fmoc-FF, suggesting that the amount of Fmoc-FF deeply affects the rigidity. Otherwise, a progressive decrease of tan δ with the increase of the Fmoc-FF component was detected. This suggests possible cooperative interaction between the Fmoc-FF component and PNA. This interaction may affect the mesh size, with a progressive increase in the elastic component’s rheological response [32]. Indeed, *G*′ values rise as the tripeptide/Fmoc-FF ratio increases and with the addition of PNA molecules, indicating a corresponding increase in the stiffness of the filled hydrogels. This effect is likely due to the gelation properties of Fmoc-FF, which reassembles into denser networks within the matrices, resulting in the formation of more rigid structures [33,34].

### 2.5. Stability Assessment of Hydrogels

The stability of empty and filled HGs was evaluated over time (up to 7 days) by incubating them in the Ringer’s solution at 40 °C and by further evaluating their weight loss [35]. The obtained results are reported in Table 5 for empty HGs and Table 6 for PNA-filled HGs. The values expressed by ΔW(%), indicate a low weight loss for all the hydrogels (<4.0%), indicating a high level of stability.

### 2.6. Swelling Rate Analysis

Swelling is a specific characteristic of gelated matter. Macroscopically, swelling represents the volumetric growth of a porous network, resulting in an increase in weight due to water entrapment. For all matrices tested, the swelling kinetics were studied for 24 h by evaluating the swelling ratios (q), reported as a percentage and correlated with weight gain (see Figures S8 and S9). All samples reached a plateau around 4 h, and after 24 h the swelling ranged between 34.8% and 38.7% (Table 7 and Table 8). As expected for multi-component matrices, the *q*-values are higher than those of the pure Fmoc-FF hydrogel (29%). No substantial differences are visible between the samples. A slight increase in swelling capacity was observed in PNA-loaded hydrogels, suggesting a role of this component in water retention.

### 2.7. Multicomponent PNA-Hydrogels’ Secondary Structure Characterization

To identify and eventually compare the occurrence of possible changes in the architecture of multicomponent hydrogels combined with PNA molecules, structural assessments were performed via CD, FT-IR, ^1^H-NMR, and SEM.

#### 2.7.1. Circular Dichroism Spectroscopy (CD)

**Figure 3:**
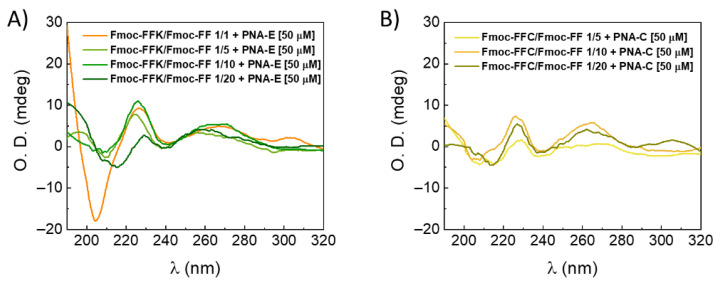
CD spectra (range 190–320 nm) of (**A**) Fmoc-FFK/Fmoc-FF (1.0 wt%) at different *w*/*w* ratios (1/1, 1/5, 1/10, and 1/20) with PNA-E [50 µM] and (**B**) Fmoc-FFC/Fmoc-FF (1.0 wt%) at different *w*/*w* ratios (1/5, 1/10, and 1/20) together with PNA-C [50 µM].

The secondary structure adopted by peptides within the hydrogel matrices was examined using CD measurements and compared with previously reported structures for standalone mixed multicomponent hydrogels [12,13]. Upon evaluating Figure 3A, it is possible to assess that there are no noteworthy distinctions between the tested samples and the CD profiles of the hydrogels without PNA. For each of them, π→π* transitions are responsible for the detection of a positive band detectable around 195 nm and a negative band centered between 205 and 210 nm [36,37]. Additionally, a signal around 230 nm is indicative of a super-helical organization of the phenylalanine residues, as previously evidenced for pristine Fmoc-FF hydrogels [8]. The broad band at 270–280 nm indicates that this arrangement causes the fluorenyl moieties in the hydrogels to have a helical-like orientation [38]. Comparing the CD spectra, the signal of the hydrogels with PNAs trapped inside is reduced. This is supported by evidence showing that the PNA component displays little to no dichroic signal (Figure S10). Control spectra were also recorded for the peptides Fmoc-FFK and Fmoc-FF at a concentration of 1 wt% (see Figure S11).

#### 2.7.2. Fourier Transform Infrared Spectroscopy (FT-IR)

**Figure 4:**
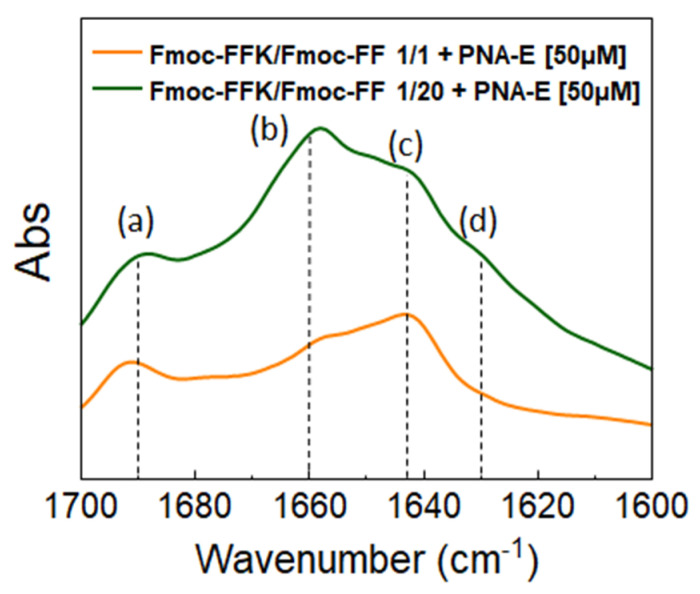
FT-IR absorbance spectra in the amide I region (range 1700–1600 cm^−1^) of Fmoc-FFK/Fmoc-FF (1.0 wt%) at the two most extreme *w*/*w* ratios (1/1, 1/20) with PNA-E [50 µM] and the identified peaks at (a) 1690 cm^−1^, (b) 1660 cm^−1^, (c) 1642 cm^−1^, and (d) 1630 cm^−1^.

FT-IR spectroscopy analysis was also used to further characterize the structural organization of peptides inside the mixed hydrogels filled with PNA molecules. Figure 4 collects the amide I region (range 1700–1600 cm^−1^) of mixed HGs combined with PNA molecules dissolved in water, presented as an absorbance spectrum. Examining the FT-IR spectra reported, three main peaks can be distinguished at ~1690 (a), 1642 (c), and 1630 cm^−1^ (d), attributable to β-sheet conformations [39,40]. The presence of a peak at 1660 cm^−1^ (b) refers to minor α-helical-like structuring, which is less intense as the Fmoc-FFK component within the hydrogels increases [41]. The formulations under study represent the two extremes in terms of Fmoc-FFK concentration. No noteworthy changes involving the secondary structure were found, thus suggesting that the arrangement is the same for 1/5 and 1/10 *w*/*w* ratios as well. The FT-IR spectrum obtained for the system containing cysteine shows the same behavior as the system shown below, thus confirming the same structuring of the PNA-filled hydrogel. The control spectra of PNA-E and PNA-C alone at a concentration of 50 µM are shown in the Supplementary Information as Figure S12A,B. To ensure data completeness, the FTIR spectra collected over the full mid-infrared range (4000–400 cm^−1^) of Fmoc-FFK/Fmoc-FF 1/1 + PNA-E and Fmoc-FFK/Fmoc-FF 1/20 + PNA-E were included in the Supplementary Materials (Figures S13 and S14, respectively).

#### 2.7.3. Peptides and PNAs ^1^H-NMR Spectroscopy

**Figure 5:**
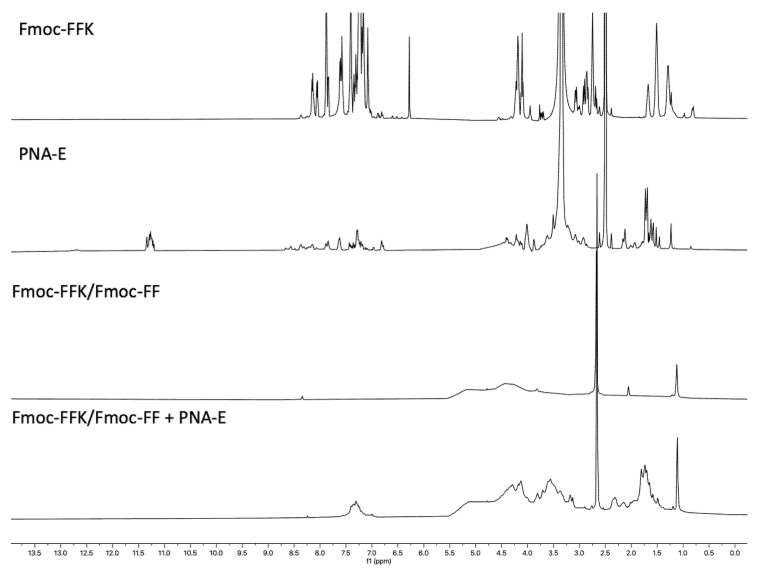
^1^H-NMR spectra of Fmoc-FFK, PNA-E, Fmoc-FFK/Fmoc-FF, and Fmoc-FFK/Fmoc-FF+PNA-E hydrogel in DMSO-d_6_.

**Figure 6:**
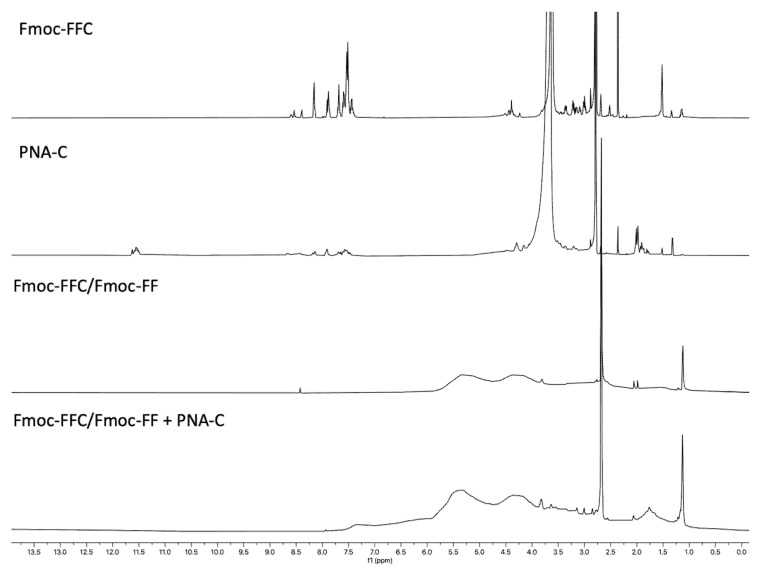
^1^H-NMR spectra of Fmoc-FFC, PNA-C, Fmoc-FFC/Fmoc-FF, and Fmoc-FFC/Fmoc-FF+PNA-C hydrogel in DMSO-d_6_.

Proton NMR spectra at 298 K were recorded for the pure peptides (Figures S15 and S16) and PNAs (Figure S17 and Table S1), as well as for the hydrogels resulting from their combination (Figure 5 and Figure 6). While the pure products were thoroughly characterized, the same level of characterization could not be achieved for the hydrogels using this technique. Liquid NMR proved unsuitable for characterizing hydrogels due to their high viscosity and semi-solid nature. The restricted molecular motion in gels leads to broad, poorly resolved signals, and their longer relaxation times result in low signal-to-noise ratios. However, one key observation that confirmed the encapsulation of PNA in the hydrogel for both samples was the appearance of new signals that were not present in the hydrogel alone (Fmoc-FFK/Fmoc-FF and Fmoc-FFC/Fmoc-FF). These signals, located in the regions between 7.5 and 7 ppm and from 5 to 1.5 ppm, are indicative of the successful encapsulation of the PNA, providing clear evidence of its incorporation into the hydrogel matrix (Figure 5 and Figure 6). Unfortunately, the assignment of the signals was not possible due to the reasons explained above.

#### 2.7.4. Fluorescence Spectroscopic Assay

**Figure 7:**
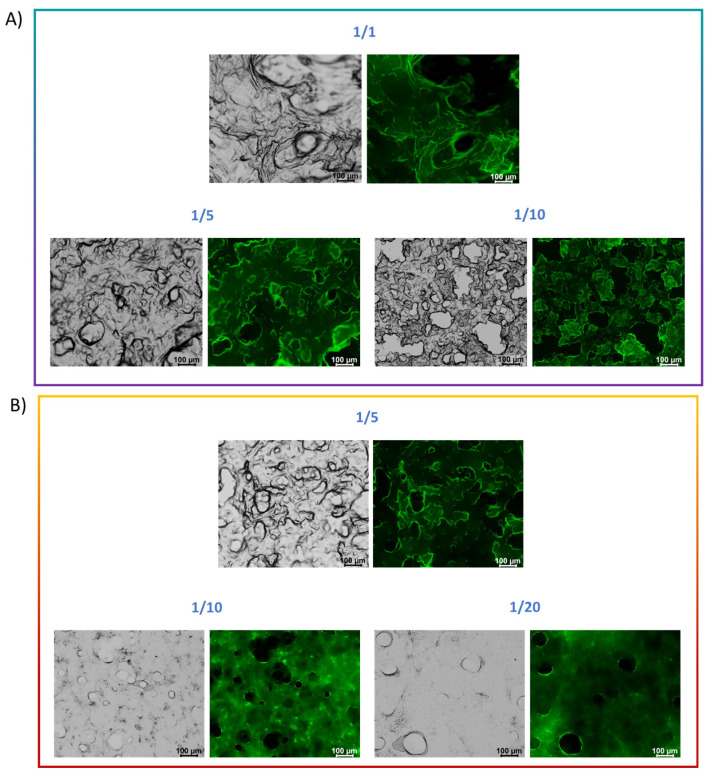
Fluorescence images of multicomponent xerogels mixed with PNA-FITC solution [25 µM] under a bright field and in the green fluorescent protein spectral region (λ_exc_ = 488 nm). (**A**) Fmoc-FFK/Fmoc-FF (1.0 wt%) at different *w*/*w* ratios (1/1, 1/5, and 1/10) with PNA-E* [25 µM]; (**B**) Fmoc-FFC/Fmoc-FF (1.0 wt%) at different *w*/*w* ratios (1/5, 1/10, and 1/20) together with PNA-C* [25 µM]. Scale bars are 100 μm for all the pictures.

This assay sheds light on the localization of labeled probes trapped in hydrogels, such as the PNA molecules [42]. The spectroscopic assay was performed on xerogels, prepared by drying preformed gels combined with PNAs [25 µM] on a clean glass slide. This PNA concentration was selected as the previously tested concentration [50 µM] exhibited a high background signal, hindering accurate visualization of the samples in fluorescence imaging. The detected fluorescence signal originates from the FITC fluorophore covalently attached to the main chain of the PNA, which reduces the steric hindrance on the molecule. The fluorescence images indicate a homogeneous distribution of PNA molecules throughout the three-dimensional hydrogel structure (Figure 7). Moreover, the pores observed in the gels form during the drying process, a common phenomenon when preparing hydrogels for microscopy, due to the dehydration caused by drying.

#### 2.7.5. SEM Analysis of Hydrogel Morphology

**Figure 8:**
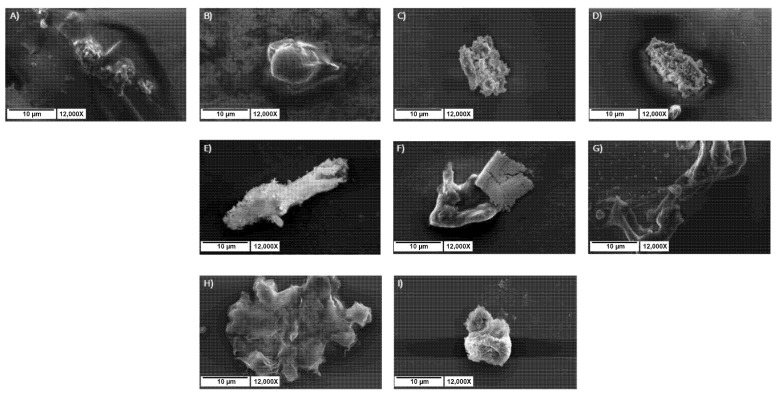
Selected micrographs of multicomponent xerogels mixed with [50µM] PNA: Fmoc-FFK/Fmoc-FF at (**A**) 1/1, (**B**) 1/5, (**C**) 1/10, and (**D**) 1/20 *w*/*w* ratios; Fmoc-FFC/Fmoc-FF at (**E**) 1/5, (**F**) 1/10, and (**G**) 1/20 *w*/*w* ratios; PNA-E alone (**H**) and PNA-C alone (**I**). Scale bars: 20 μm for all the pictures. Scale bar is 10 μm, and the magnification is 12,000×.

SEM was employed to assess the topography and morphology of the supramolecular components constituting the hydrogel matrices, along with the PNAs encapsulated within them. Figure 6 displays representative SEM photomicrographs, demonstrating that each sample is free of long, unbranched fibers, a type of entanglement frequently seen in peptide hydrogels [43]. Indeed, earlier characterizations performed solely on peptide hydrogels verified this underlying structure [12,13]. On the other hand, by adding PNAs into the hydrogels’ matrix, a more rounded shape is noticeable, possibly due to the presence of trapped PNA molecules within the peptides’ network [44]. The two sequences of PNA used in this study were investigated without the hydrogels under the scanning electron microscope as well. From the SEM micrographs in Figure 8H,I, it can be observed that even the PNA alone has a propensity to self-assemble into large amorphous aggregates.

### 2.8. Diffusion Studies of PNAs in Hydrogel Matrices

**Figure 9:**
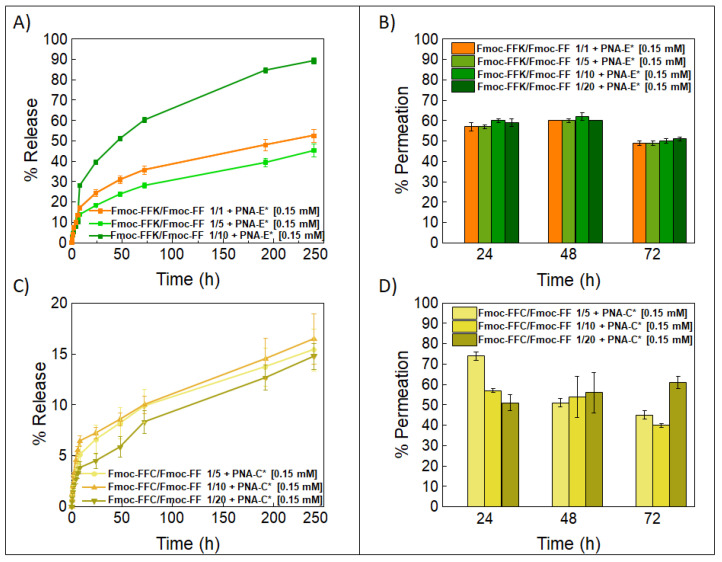
The release kinetics of PNA-E* and PNA-C* solutions [0.15 mM] over 10 days are reported (**A**,**C**). This solution was used to formulate 400 µL of the Fmoc-FFK and Fmoc-FFC hydrogels at different *w*/*w* ratios together with Fmoc-FF. Permeation assays were carried out by incubating Fmoc-FFK and Fmoc-FFC hydrogels (400 µL) at different weight ratios with Fmoc-FF (1/1, 1/5, 1/10, and 1/20) with 800 µL PNA-E* and PNA-C* solutions [0.15 mM] over 72 h (**B**,**D**).

The capability of multicomponent hydrogels to retain drug molecules and thus serve as reservoirs for controlled drug release was tested by encapsulating PNA molecules within the matrices and evaluating the release over time. Two systems were taken under consideration for these studies: Fmoc-FFC HGs coupled with PNA-C* and Fmoc-FFK HGs interacting with PNA-E*. The release profile of PNA-FITC molecules from the hydrogels was efficiently monitored using UV–Vis spectroscopy. During the rehydration phase of the solvent switch formulation procedure, a total of [0.15 mM] PNA-FITC was retained within the hydrogel networks. Following quantitative PNA-FITC loading, no syneresis was seen. Figure 9A,C report the PNA release kinetics over a period of 10 days. The Fmoc-FFK/Fmoc-FF 1/20 formulation was not included in the current investigation as the hydrogel precipitated at the abovementioned PNA-E* concentration, thus preventing the appropriate gel formation. For PNA-E* loaded hydrogels prepared by mixing Fmoc-FFK and Fmoc-FF at 1/1 and 1/5 *w*/*w* ratios, similar release behaviors can be observed at 53% and 45%, respectively. On the contrary, the release significantly increases (up to 90%) in the Fmoc-FFK/Fmoc-FF 1/10 HG.

Regarding the system involving Fmoc-FFC and PNA-C* hydrogels, the three gels at different weight ratios (1/5, 1/10, and 1/20) displayed a lower release rate compared to PNA-E+Fmoc-FFK/Fmoc-FF (~15%), over the same period. The PNA release does not seem to be related to either the mechanical or swelling properties. This different behavior likely reflects fundamental differences in the molecular interaction mechanisms underlying the two systems. Qualitative assessment of the slope of PNA release profiles from hydrogels provides valuable insights into the underlying release mechanisms (Figure S18). In Fmoc-FFK/PNA-E* hydrogels, electrostatic interactions between Lys and glutamic acid Glu residues enable reversible binding of the PNA, resulting in higher release rates, particularly under conditions with variable pH or ionic strength. This makes such hydrogels especially suitable for therapeutic applications requiring rapid or localized release, such as drug delivery in tumor microenvironments, where acidic conditions may enhance payload release [45].

In contrast, the Fmoc-FFC/PNA-C* system is based on the formation of covalent disulfide bonds between Cys residues. In this case, the release mechanism depends on the presence of reducing conditions, which are required to cleave the disulfide linkages and gradually liberate the encapsulated PNA. Therefore, this type of hydrogel is more appropriate for long-term delivery strategies, such as chronic disease management or implantable drug reservoirs, where sustained and controlled release over extended periods is desired [46].

Permeation studies with PNA-FITC derivatives were also carried out on the empty HGs using the same concentrations of PNA-FITC solutions. Permeation was monitored at three time points (24, 48, and 72 h), and the results are shown in Figure 9B,D. Despite the different weight ratios between the lysine-containing hydrogels, the former system showed a similar permeation rate (~50% after 72 h) for all samples. From the comparison of the permeation rate over time, it seems there is a slight, not significant, decrease in the permeation over time. Instead, Fmoc-FFC mixed HGs showed a permeation rate of 45%, 40%, and 60% for the 1/5, 1/10, and 1/20 *w*/*w* ratios, respectively. In this case, a variation of the PNA-C* molecule can be observed as a function of both the time and the HG composition. The overall findings highlight the possibility that stiffness and electrostatic interactions are not the only variables that affect drug retention.

One reasonable explanation is that the fluorophore’s propensity to escape or to be retained from HG may be influenced by the matrix’s varying hydrophilic and hydrophobic properties, which result in varying degrees of water accessibility.

## 3. Conclusions

This study demonstrates the potential of peptide-based multicomponent hydrogels for the efficient loading and controlled release of peptide nucleic acids in drug delivery applications. Two distinct loading strategies were explored: non-covalent encapsulation via electrostatic Lys–Glu interactions and covalent incorporation through Cys–Cys disulfide bonds. Hydrogels were polymerized directly in the presence of PNAs, allowing the systematic optimization of formulation by varying the ratio of hydrogel components. Among all tested conditions, the 1:10 Fmoc-FFX/Fmoc-FF ratio provided the best compromise between structural integrity and release performance. These formulations showed excellent physicochemical stability, with minimal swelling (ΔW < 4%), high water retention, and significant mechanical strength (G′ > 19,000 Pa for FFK-based and >22,000 Pa for FFC-based systems). The two loading strategies resulted in distinct release profiles over a 10-day period. Electrostatic loading enabled a faster and higher release (up to 90%), suitable for dynamic or acidic microenvironments such as tumor tissues, whereas covalent bonding led to a lower (up to 15%) and sustained release, ideal for chronic treatments or implantable drug delivery systems. This behavior was further supported by qualitative differences in the slopes of the release curves. These findings underscore the versatility and tunability of peptide-based hydrogels as adaptable platforms for PNA delivery.

## 4. Materials and Methods

### 4.1. Chemicals and Reagents

Protected *N_α_*-Fmoc-amino acids, coupling reagents and Rink amide MBHA (4-methylbenzhydrylamine) resin were purchased from Calbiochem-Novabiochem (Läufelfingen, Switzerland). Fmoc-FF peptide was bought from Bachem (Bubendorf, Switzerland). *N*-(2-((((9H-fluoren-9-yl)methoxy)-carbonyl)amino)ethyl)-*N*-(2-(5-methyl-2,4-dioxo-3,4-dihydropyrimidin-1(2H)-yl)acetyl)glycine (Fmoc-PNA-T-OH) as well as the spacer [2-(2-(Fmoc-amino)ethoxy)ethoxy]acetic acid (Fmoc-PEG_2_-OH) were purchased from LGC Biosearch Technologies™ (Bellshill, Scotland, UK). Dithiothreitol (DTT) from Merck (Milan, Italy). All other chemical products are commercially available from Merck (Milan, Italy), Fluka (Bucks, Switzerland), or LabScan (Stillorgan, Ireland) and, unless stated otherwise, were used as delivered by the companies.

### 4.2. PNA and Peptides Synthesis, Purification, and Analysis

PNA sequences (Table 1) were synthesized using the 9-fluorenylmethoxycarbonyl (Fmoc) solid-phase strategy, following the protocol reported elsewhere [15]. The last two coupling processes were carried out for two hours each prior to the detachment from the resin to covalently bind two spacer molecules (Fmoc-PEG_2_-OH) at the *N*-terminus on the PNA-C* and PNA-E* main chains. At the end of the synthesis, PNA sequences were left under stirring for 3 h in a solution of Trifluoroacetic acid (TFA)/triisopropylsilane (TIS)/1,2-ethanedithiol/H_2_O (92.5/2.5/2.5/2.5 *v*/*v*/*v*/*v*), to promote the detachment from the resin. Then, the PNAs underwent a precipitation step, performed in cold diethyl ether and after this, three cycles of lyophilization. The raw product was purified by semipreparative HPLC analyses by Jasco (Easton, MD, USA) Plus pump equipped with a Jasco UV-2075 Plus UV detector using a 10 × 250 mm C-18 reverse-phase column (particle size 5 µm) (Merck Millipore Billerica, MA, USA) eluted with a linear gradient of CH_3_CN containing 0.1% (*v*/*v*) TFA in H_2_O containing 0.1% (*v*/*v*) TFA (from 0 to 100% in 45 min, flow 1.2 mL min^−1^). The purified PNA was lyophilized using a SCANVAC Cool Safe 55 freeze-dryer (ScanLaf A/S, Lynge, Denmark) by setting the freezing temperature at –65 °C, vacuum pressure at 0.01 mbar, and heating temperature at +100 °C. After the lyophilization process, the samples were dissolved in pure water estimating the concentration by UV analyses by the Jasco V-530 spectrophotometer (λ = 220–310 nm, 400 nm min^−1^ scanning speed, 2.0 nm bandwidth; ε _(260)_ = 35,200 cm^−1^M^−1^ for PNA-C and PNA-E; ε _(260)_ = 48,900 cm^−1^M^−1^ for PNA-C* and PNA-E*. The products were characterized by an ESI-MS Applied Biosystems 4000 QTRAP mass spectrometer (Figure S19). Fmoc-FFK and Fmoc-FFC tripeptides were synthesized according to standard solid-phase peptide synthesis (SPPS) procedures using the Fmoc/*t*Bu strategy [47] as previously described [12,13].

### 4.3. Formulation of Hydrogels

Samples and peptide-based hydrogels were prepared by weight using dimethyl sulfoxide (DMSO) and double-distilled water. All mixed hydrogels were formulated at a concentration of 1.0 wt% (10 mg·mL^−1^) via the DMSO/H_2_O solvent-switch method by using different *w*/*w* ratios of the two tripeptides, Fmoc-FFC and Fmoc-FFK, and of the aromatic dipeptide Fmoc-FF. The examined ratios for mixed hydrogels were 1/1, 1/5, 1/10, and 1/20 *w*/*w*. Stock solutions of each peptide were prepared in DMSO (100 mg·mL^−1^), then mixed, vortexed, and subsequently rehydrated with PNA solutions previously quantified via UV–Vis spectroscopy as described above and dissolved in doubly distilled water, filtered with a 22 µm filter diameter. During the rehydration step, the mixtures were additionally stirred for ~3 s to promote the homogeneity of the samples. The formation of hydrogels was macroscopically assessed via an inverted test tube. Furthermore, 150 µL of all samples were analyzed as a function of time during the opaque-limpid transition phase using multiwell 96 plates in the long range (600 nm) to determine the gelation times of the hydrogels following the addition of PNA molecules. The instrument employed was a Victor Nivo PerkinElmer plate reader.

### 4.4. Rheology

Rheological measurements of freshly preformed PNA-loaded hydrogels (500 µL) at 1.0 wt% were performed with a rotational controlled-stress rheometer (Malvern Kinexus, London, UK) equipped with a 1.5 cm diameter flat-plate geometry (PU20-PL61). Each analysis was carried out in accordance with the previously mentioned setup [12,13]. The rheological profiles, reported in Pascal (Pa), were plotted as storage or elastic modulus (*G*′) and shear loss or viscous modulus (*G*″). Each measurement has been performed in duplicate.

### 4.5. Hydrogel Stability Studies

The determination of the hydrogel degradation profile with or without the presence of PNAs was performed by an in vitro stability assay to evaluate the percentage of weight loss of the matrices. Freshly formed 1.0 wt % hydrogels (300 μL) were weighed (W_0_) and then incubated at 40 °C with 900 μL of Ringer’s solution (8.6 mg of NaCl, 0.30 mg of KCl, and 0.33 mg of CaCl_2_) [48]. The Ringer’s solution was taken out after seven days, and the hydrogels were once more weighed (W_t_). The following formula was used to represent the degree of degradation as a percentage ratio (ΔW) between the hydrogel weight before (W_0_) and after (W_t_) the treatment

(1) $$\Delta W=\left(1-\frac{Wt}{W0}\right)\times 100$$

The experiment has been performed in duplicate.

### 4.6. Swelling Kinetics

The swelling kinetics of the matrix were evaluated over 24 h at 25 °C. Swelling ratios (*q*), expressed as a percentage, were evaluated by adding 600 μL of double-distilled water to 300 μL of freshly prepared hydrogel (1.0% wt). After removing excess water, *q* was estimated for each time point using Equation (2). *Ws* and *Wd* indicate the weight of the fully swollen hydrogel obtained after removal of excess water and lyophilized samples, respectively.

(2) $$q=\left(\frac{Ws}{Wd}-1\right)\times 100$$

### 4.7. Circular Dichroism (CD) Spectroscopy

CD spectra of all the peptide-based multicomponent materials in combination with PNA solutions 50µM were recorded in the 320–190 nm spectral region. Each sample was allocated in a 0.2 mm quartz cell equilibrated at 25 °C. Measurements were performed on a Jasco J-1500-150 spectropolarimeter equipped with a Jasco MCB-100 Mini Water Circulation Bath thermal controller unit (Peltier device), as previously described [12,13]. All the HGs were investigated in combination with PNA solutions.

### 4.8. Fourier Transform Infrared Spectroscopy (FT-IR)

FT-IR spectra of all the 1.0 wt% peptide-based hydrogels in combination with 50 µM PNA solutions were performed on a Jasco FT/IR 4100 spectrometer (Easton, MD, USA) as previously described [12]. After collection in transmission mode, amide I absorbance profiles (in the 1700–1600 cm^−1^ region) were automatically returned as emissions by the instrument’s integrated software, namely secondary structure analysis (SSE) software for Infrared Interpretation and modeling of proteins, version 2.15B.

### 4.9. ^1^H-NMR Spectroscopy

NMR experiments were recorded on an Avance Neo (600 MHz) spectrometer equipped with an HCN triple resonance CryoProbe (Bruker-Biospin, Billerica, MA, USA) at 298 K. Chemical shifts (δ) are reported in ppm downfield from the residual solvent peak, whereas coupling constants (*J*) are stated in Hz. NMR data processing was performed with MestReNova 14.2.0 software (Mestrelab Research, Santiago de Compostela, Spain). NMR samples of pure Fmoc-FFK (3 mg), Fmoc-FFC (3 mg), PNA-E (1.5 mg), and PNA-C (1.5 mg) were obtained by dissolving the samples in 500 µL DMSO-d6. NMR samples of Fmoc-FFK/Fmoc-FF+PNA-E and Fmoc-FFC/Fmoc-FF+PNA-C hydrogels were prepared directly in NMR tubes using D_2_O/DMSO-d_6_ (2:1 *v*/*v*) and analyzed after gelation. All spectra in aqueous buffers were referenced to the residual H_2_O signal (4.78 ppm at 298 K).

^1^H-NMR of Fmoc-FFK (600 MHz, DMSO-d_6_, Figure S11) (chemical shifts in δ, DMSO-d_6_ as internal standard 2.55) δ 8.13 (dd, J = 15.6, 6.9 Hz, 1H), 8.05 (d, J = 8.1 Hz, 1H), 7.86 (dd, J = 21.6, 7.2 Hz, 2H), 7.65–7.55 (m, 2H), 7.41 (q, J = 7.1 Hz, 2H), 7.24 (dd, J = 13.6, 6.2 Hz, 6H), 7.08 (s, 1H), 6.28 (s, 0H), 4.59–4.49 (m, 1H), 4.25–4.15 (m, 2H), 4.14–4.06 (m, 1H), 3.04 (ddd, J = 33.0, 13.9, 4.6 Hz, 1H), 2.91 (dd, J = 14.0, 3.7 Hz, 1H), 2.78–2.65 (m, 3H), 1.68 (h, J = 6.3 Hz, 1H), 1.51 (dq, J = 15.7, 7.0 Hz, 3H), 1.28 (tt, J = 24.6, 12.2 Hz, 2H).

^1^H-NMR of Fmoc-FFC (600 MHz, DMSO-d_6_, Figure S12) δ 8.33–8.28 (m, 1H), 8.24 (dd, J = 23.4, 7.7 Hz, 2H), 8.11 (d, J = 8.1 Hz, 2H), 7.87 (dt, J = 9.9, 4.9 Hz, 8H), 7.60 (ddt, J = 20.8, 14.9, 7.4 Hz, 12H), 7.40 (p, J = 7.7 Hz, 9H), 7.30 (dt, J = 20.3, 7.7 Hz, 6H), 7.24–7.18 (m, 38H), 7.16–7.12 (m, 2H), 4.59–4.55 (m, 3H), 4.33 (q, J = 7.0 Hz, 3H), 4.22 (td, J = 9.6, 4.2 Hz, 4H), 4.18–4.03 (m, 11H), 3.94 (d, J = 17.8 Hz, 1H), 3.18–3.10 (m, 1H), 3.08–3.01 (m, 2H), 2.96–2.77 (m, 11H), 2.72 (ddd, J = 13.7, 10.6, 7.0 Hz, 6H), 2.26 (dt, J = 33.1, 8.0 Hz, 2H), 2.07 (s, 16H), 1.29–1.22 (m, 12H), 1.05 (t, J = 7.0 Hz, 1H), 0.86 (dt, J = 13.9, 7.3 Hz, 3H).

^1^H-NMR of PNA-E (600 MHz, DMSO-d_6_, Figure S13) δ 12.68 (s, 1H), 11.36–11.19 (m, 5H), 8.15 (d, J = 10.5 Hz, 1H), 7.85 (s, 1H), 7.63–7.60 (m, 2H), 7.45–7.40 (m, 1H), 7.28 (d, J = 6.2 Hz, 3H), 6.81 (s, 1H), 4.67 (dd, J = 16.8, 5.8 Hz, 3H), 4.53–4.45 (m, 1H), 4.40 (d, J = 14.0 Hz, 2H), 4.23–4.13 (m, 2H), 4.05–3.98 (m, 6H), 3.90–3.86 (m, 2H), 3.62 (s, 2H), 3.51 (d, J = 4.7 Hz, 2H), 3.08 (s, 2H), 2.93 (dd, J = 12.9, 6.5 Hz, 2H), 2.14 (dt, J = 22.9, 8.1 Hz, 3H), 1.72 (dd, J = 18.4, 8.5 Hz, 8H), 1.52 (s, 1H), 1.46 (d, J = 3.9 Hz, 1H), 1.23 (s, 1H).

^1^H-NMR of PNA-C (600 MHz, DMSO-d_6_, Figure S13) δ 11.35–11.17 (m, 1H), 7.50–7.16 (m, 1H), 4.67 (d, J = 26.9 Hz, 1H), 4.61–4.35 (m, 1H), 4.02 (d, J = 23.0 Hz, 1H), 3.40–3.35 (m, 191H), 1.76–1.67 (m, 2H), 1.62 (td, J = 21.0, 7.3 Hz, 1H).

### 4.10. Fluorescence Spectroscopy

The aggregation behavior of mixed hydrogels in combination with PNA-FITC solutions was assessed using a spectroscopic assay. Hydrogels (200 μL) were prepared according to the solvent-switch method, as reported above, by using PNA solutions [25 μM]. After gel formation, ~150 μL of the samples were deposited on a clean coverslip glass and left to dry overnight. The dried stained films, or xerogels, were observed under bright-field illumination and in the spectral region of the FITC fluorophore (λ_exc_ = 488 nm, λ_em_= 520 nm) [49]. Scale bars were acquired with a magnification of 100 μm for all the pictures. Fluorescence images were taken with a Leica MICA micro-hub fluorescence microscope equipped with a 10× objective.

### 4.11. Scanning Electron Microscopy (SEM)

Morphological analysis of xerogels was carried out by field-emission SEM on a Phenom ProX instrument (Alfatest, Milan, Italy) as previously described [12,13]. The analyses were carried out on samples previously prepared by drop-casting 40 μL of each multicomponent peptide hydrogel combined with PNAs [50 µM] on an aluminum pin stub, whereas 20 μL was used for PNAs solutions [50 µM]. Further details: scale bars 10 µm; Magnification 12,000×.

### 4.12. Release and Permeation Studies of PNAs

Using the previously described method, conical tubes containing PNA-FITC encapsulating HGs (400 µL) were prepared. Each peptide’s DMSO solution was rehydrated using a PNA-FITC water solution at a concentration of 0.00015 mol L^−1^. Using UV–Vis spectroscopy, the concentration of PNA solutions (ε_490_ = 48,900 L·cm^−1^) was quantitatively measured. Each hydrogel with PNA-K was covered with a total of 800 µL of water, while the hydrogel with PNA-C was covered with 800 µL of a 20 mM DTT water solution [15]. At predefined intervals, 400 µL of this solution was withdrawn and replaced with 400 µL of fresh water. The amount of PNAs in each fraction was followed over 10 days and estimated via UV–vis spectroscopy using a NanoPhotometer^®^ NP80 Nanodrop (Implen, Munich, Germany). The results were then expressed as a percentage of the ratio between the initial amount that was encapsulated and the released PNAs. Permeation assays were performed by incubating 400 µL of preformed hydrogels in 1.5 mL conical tubes with 800 µL of PNA-FITC solution 0.00015 mol L^−1^. The hydrogels’ permeation was followed over 72 h.

## Figures and Tables

**Table 1:** Sequences of PNAs used in this study. PNA bases are in lowercase letters.

Sample	Sequence (*C* → *N*)	Chemical Formula	M.W. calc. (a.m.u.)	M.W. det. (a.m.u.)	Rt (min)
PNA-E	$E-t_{4}-{NH}_{2}$	C_49_H_65_N_17_O_20_	1211.46	1211.6	11.2
PNA-E*	$E-t_{4}-({PEG2)}_{2}-FITC$	C_82_H_98_N_20_O_31_S	1891.64	1891.7	16.4
PNA-C	$C-t_{4}-{NH}_{2}$	C_47_H_64_N_18_O_17_S	1185.44	1185.5	11.6
PNA-C*	$C-t_{4}-{\left(PEG2\right)}_{2}-FITC$	C_80_H_97_N_21_O_28_S_2_	1865.63	1865.7	16.2

**Table 2:** Chemical formula and theoretical and experimentally found molecular weights (M.W.s) of the investigated peptides.

Peptide	Chemical Formula	M.W. calc. (a.m.u.)	M.W. det. (a.m.u.)
Fmoc-FFC	C_36_H_36_N_4_O_5_S	636.7	636.3
Fmoc-FFK	C_39_H_42_N_4_O_6_	662.7	662.5

**Table 3:** Characterization of mixed hydrogels at different *w*/*w* ratios. Gelation times (G*t*) of mixed hydrogels with and without the PNA addition.

System (HGs)	Ratio with Fmoc-FF (*w*/*w*)	Gelation Time (min)	Gelation Time of Solely HGs (min)
Fmoc-FFK/Fmoc-FF + PNA-E [50µM]	1/1 1/5 1/10 1/20	26 112 164 34	10 78 138 216
Fmoc-FFC/Fmoc-FF + PNA-C [50µM]	1/5 1/10 1/20	80 25 10	75 4 2

**Table 4:** Rheological analysis of hydrogels loaded with PNAs. Reported data are the storage modulus (*G*′), loss modulus (*G*″), and tan δ (*G*′/*G*″).

*Fmoc-FFK/Fmoc-FF* + PNA-E				*Fmoc-FFC/Fmoc-FF* + PNA-C			
Ratio with Fmoc-FF	G′ (Pa)	G″ (Pa)	tan δ	Ratio with Fmoc-FF	G′ (Pa)	G″ (Pa)	tan δ
1/1	140	20	0.143				
1/5	19320	1770	0.0920	1/5	648	112	0.173
1/10	6243	444	0.0710	1/10	6941	1019	0.147
1/20	8711	486	0.0557	1/20	22471	2371	0.105

**Table 5:** Stability test of empty hydrogels.

*Fmoc-FFK/Fmoc-FF*				*Fmoc-FFC/Fmoc-FF*			
Ratio with Fmoc-FF	*W*_0_ (g)	*W_t_* (g)	Δ*W* (%)	Ratio with Fmoc-FF	*W*_0_ (g)	*W_t_* (g)	Δ*W* (%)
1/1	1.3438	1.3263	1				
1/5	1.3411	1.3200	2	1/5	1.3362	1.3229	1
1/10	1.3320	1.3290	0	1/10	1.3429	1.3298	1
1/20	1.3363	1.2979	3	1/20	1.3417	1.3393	0

**Table 6:** Stability test of hydrogels loaded with PNAs.

*Fmoc-FFK/Fmoc-FF + PNA-E*				*Fmoc-FFC/Fmoc-FF + PNA-C*			
Ratio with Fmoc-FF	*W*_0_ (g)	*W_t_* (g)	Δ*W* (%)	Ratio with Fmoc-FF	*W*_0_ (g)	*W_t_* (g)	Δ*W* (%)
1/1	1.3023	1.2849	1				
1/5	1.3280	1.3089	1	1/5	1.2802	1.2244	4
1/10	1.3266	1.3034	2	1/10	1.2927	1.2401	4
1/20	1.3306	1.2869	3	1/20	1.3015	1.2519	4

**Table 7:** *q*-values (%) at 24 h for empty matrices.

Sample (Empty HGs)	Ratio with Fmoc-FF	q (%)
Fmoc-FFK/Fmoc-FF	1/1	36.1
	1/5	36.8
	1/10	35.0
	1/20	34.8
Fmoc-FFC/Fmoc-FF	1/5	38.7
	1/10	37.2
	1/20	36.2

**Table 8:** *q*-values (%) at 24 h for PNA-loaded matrices.

Sample (Loaded HGs)	Ratio with Fmoc-FF	q (%)
Fmoc-FFK/Fmoc-FF + PNA-E	1/1	37.7
	1/5	37.7
	1/10	37.3
	1/20	37.9
Fmoc-FFC/Fmoc-FF + PNA-C	1/5	36.7
	1/10	36.9
	1/20	37.9

## Data Availability

The original contributions presented in this study are included in the article/Supplementary Material. Further inquiries can be directed to the corresponding authors.

## Supplementary Materials

The following supporting information can be downloaded at: https://www.mdpi.com/article/10.3390/gels11050367/s1, Figure S1: Physicochemical characterization of PNA-E; Figure S2: Physicochemical characterization of PNA-E*; Figure S3: Physicochemical characterization of PNA-C; Figure S4: Physicochemical characterization of PNA-C*; Figure S5: Inverted test tubes of each peptide alone (Fmoc-FF, Fmoc-FFK, and Fmoc-FFC) at 1.0 wt%; Figure S6: UV–Vis profiles of PNA-functionalized Fmoc-FFX/Fmoc-FF mixed hydrogels; Figure S7: Rheological analysis of PNA-functionalized Fmoc-FFX/Fmoc-FF mixed hydrogels; Figure S8: Swelling kinetics for Fmoc-FFK/Fmoc-FF and Fmoc-FFC/Fmoc-FF matrices; Figure S9: Swelling kinetics for Fmoc-FFK/Fmoc-FF and Fmoc-FFC/Fmoc-FF matrices containing PNA-C and PNA-E; Figure S10: CD spectra of PNA-E and PNA-C; Figure S11. CD spectra (range 320–190 nm) of (A) Fmoc-FF and (B) Fmoc-FFK at 1.0 wt%; Figure S12. FTIR spectra collected over the full mid-infrared range (4000–400 cm^−1^) of Fmoc-FFK/Fmoc-FF 1/1 + PNA-E [50 µM]; Figure S13. FTIR spectra collected over the full mid-infrared range (4000–400 cm^−1^) of Fmoc-FFK/Fmoc-FF 1/20 + PNA-E [50 µM]; Figure S14. FT-IR absorbance spectra in the amide I region of PNA-E and PNA-C; Figure S15. ^1^H-NMR spectrum of Fmoc-FFK in DMSO-d6; Figure S16. ^1^H-NMR spectrum of Fmoc-FFC in DMSO-d6; Figure S17. ^1^H-NMR spectrum of PNA-C and PNA-E in DMSO-d6; Table S1 of ¹H NMR Chemical Shifts for PNA-C and PNA-E in DMSO-d_6_; Figure S18. Release kinetics of PNA-E* and PNA-C* (0.15 mM) from peptide-based hydrogels over a 10-day period are shown in panels A and C, respectively. Dashed lines parallel to the *x*-axis are included to support qualitative assessment of the slope, highlighting differences in release profiles associated with electrostatic (PNA-E*) and covalent (PNA-C*) interactions; Figure S19. Physicochemical characterization of Fmoc-FFC-S-S-cys-PNA (1.0 EQ) via ESI mass spectrometry (Enhanced Resolution).

## References

[1] Zhang C., Yan L., Wang X., Zhu S., Chen C., Gu Z., Zhao Y. (2020). Progress, challenges, and future of nanomedicine. Nano Today.

[2] Mondal S., Das S., Nandi A.K. (2020). A review on recent advances in polymer and peptide hydrogels. Soft Matter.

[3] Karchoubi F., Afshar Ghotli R., Pahlevani H., Baghban Salehi M. (2024). New insights into nanocomposite hydrogels: A review on recent advances in characteristics and applications. Adv. Ind. Eng. Polym. Res..

[4] Binaymotlagh R., Chronopoulou L., Haghighi F.H., Fratoddi I., Palocci C. (2022). Peptide-based hydrogels: New materials for biosensing and biomedical applications. Materials.

[5] Das S., Das D. (2021). Rational design of peptide-based smart hydrogels for therapeutic applications. Front. Chem..

[6] Park S., Kim S.H., Dezhbord M., Lee E.-H., Jeon Y., Jung D., Gu S.H., Yu C., Lee S.H., Kim S.C. (2023). Cell-permeable peptide nucleic acid antisense oligonucleotide platform targeting human betacoronaviruses. Front. Microbiol..

[7] Diaferia C., Rosa E., Gallo E., Morelli G., Accardo A. (2023). Differently N-capped analogues of Fmoc-FF. Chem. Eur. J..

[8] Wang Y., Geng Q., Zhang Y., Adler-Abramovich L., Fan X., Mei D., Gazit E., Tao K. (2023). Fmoc-diphenylalanine gelating nanoarchitectonics: A simplistic peptide self-assembly to meet complex applications. J. Colloid Interface Sci..

[9] Choe R., Il Yun S. (2020). Fmoc-diphenylalanine-based hydrogels as a potential carrier for drug delivery. e-Polymers.

[10] Diaferia C., Ghosh M., Sibillano T., Gallo E., Stornaiuolo M., Giannini C., Morelli G., Adler-Abramovich L., Accardo A. (2019). Fmoc-FF and hexapeptide-based multicomponent hydrogels as scaffold materials. Soft Matter.

[11] Apostolidou C.P., Kokotidou C., Platania V., Nikolaou V., Landrou G., Nikoloudakis E., Charalambidis G., Chatzinikolaidou M., Coutsolelos A.G., Mitraki A. (2024). Antimicrobial Potency of Fmoc-Phe-Phe Dipeptide Hydrogels with Encapsulated Porphyrin Chromophores Is a Promising Alternative in Antimicrobial Resistance. Biomolecules.

[12] Giordano S., Gallo E., Diaferia C., Rosa E., Carrese B., Borbone N., Scognamiglio P.L., Franzese M., Oliviero G., Accardo A. (2023). Multicomponent peptide-based hydrogels containing chemical functional groups as innovative platforms for biotechnological applications. Gels.

[13] Gallo E., Diaferia C., Giordano S., Rosa E., Carrese B., Piccialli G., Borbone N., Morelli G., Oliviero G., Accardo A. (2023). Ultrashort cationic peptide Fmoc-FFK as hydrogel building block for potential biomedical applications. Gels.

[14] Uhlmann E., Peyman A., Breipohl G., Will D.W. (1998). PNA: Synthetic polyamide nucleic acids with unusual binding properties. Angew. Chem. Int. Ed..

[15] Falanga A.P., Cerullo V., Marzano M., Feola S., Oliviero G., Piccialli G., Borbone N. (2019). Peptide nucleic acid-functionalized adenoviral vectors targeting G-quadruplexes in the P1 promoter of Bcl-2 proto-oncogene: A new tool for gene modulation in anticancer therapy. Bioconjug. Chem..

[16] Hanvey J.C., Peffer N.J., Bisi J.E., Thomson S.A., Cadilla R., Josey J.A., Ricca D.J., Hassman C.F., Bonham M.A., Au K.G. (1992). Antisense and antigene properties of peptide nucleic acids. Science.

[17] Terracciano M., Fontana F., Falanga A.P., D’Errico S., Torrieri G., Greco F., Tramontano C., Rea I., Piccialli G., De Stefano L. (2022). Development of surface chemical strategies for synthesizing redox-responsive diatomite nanoparticles as a green platform for on-demand intracellular release of an antisense peptide nucleic acid anticancer agent. Small.

[18] MacLelland V., Kravitz M., Gupta A. (2024). Therapeutic and diagnostic applications of antisense peptide nucleic acids. Mol. Ther. Nucleic Acids.

[19] Sannigrahi A., De N., Bhunia D., Bhadra J. (2025). Peptide nucleic acids: Recent advancements and future opportunities in biomedical applications. Bioorg. Chem..

[20] Zarrilli F., Amato F., Morgillo C.M., Pinto B., Santarpia G., Borbone N., D’Errico S., Catalanotti B., Piccialli G., Castaldo G. (2017). Peptide nucleic acids as miRNA target protectors for the treatment of cystic fibrosis. Molecules.

[21] Cadoni E., Manicardi A., Madder A. (2020). PNA-Based MicroRNA Detection Methodologies. Molecules.

[22] Gaglione M., Milano G., Chambery A., Moggio L., Romanelli A., Messere A. (2011). PNA-based artificial nucleases as antisense and anti-miRNA oligonucleotide agents. Mol. Biosyst..

[23] Kim Y., Mohanty S.K. (2025). PNA functionalized gold nanoparticles on TiO_2_ nanotubes biosensor for electrochemical DNA fragment detection. Adv. Mat. Interfaces.

[24] Crisci T., Falanga A.P., Casalino M., Borbone N., Terracciano M., Chianese G., Gioffrè M., D’Errico S., Marzano M., Rea I. (2021). Bioconjugation of a PNA probe to zinc oxide nanowires for label-free sensing. Nanomaterials.

[25] Pooga M., Land T., Bartfai T., Langel Ü. (2001). PNA oligomers as tools for specific modulation of gene expression. Biomol. Eng..

[26] Berger S., Lächelt U., Wagner E. (2024). Dynamic carriers for therapeutic RNA delivery. Proc. Natl. Acad. Sci. USA.

[27] Gupta A., Bahal R., Gupta M., Glazer P.M., Saltzman W.M. (2016). Nanotechnology for delivery of peptide nucleic acids (PNAs). J. Control Release.

[28] Ambasht P.K. (2020). Use of group-specific reagents in active site functional group elucidation I: Cys, Ser, Tyr, and Trp residues. Frontiers in Protein Structure, Function, and Dynamics.

[29] Choi H., Go M., Cha Y., Choi Y., Kwon K.-Y., Jung J.H. (2017). A facile method to fabricate hydrogels from DMSO polymer gels via solvent exchange. New J. Chem..

[30] Tang C., Smith A.M., Collins R.F., Ulijn R.V., Saiani A. (2009). Fmoc-diphenylalanine self-assembly mechanism induces apparent pKa shifts. Langmuir.

[31] Katoueizadeh E., Rasouli M., Zebarjad S.M. (2021). The rheological behavior of the non-Newtonian thixotropic colloidal silica gels from sodium silicate. Mater. Chem. Phys..

[32] Stojkov G., Niyazov Z., Picchioni F., Bose R.K. (2021). Relationship between structure and rheology of hydrogels for various applications. Gels.

[33] Adams D.J., Mullen L.M., Berta M., Chen L., Frith W.J. (2010). Relationship between molecular structure, gelation behaviour and gel properties of Fmoc-dipeptides. Soft Matter.

[34] Orbach R., Mironi-Harpaz I., Adler-Abramovich L., Mossou E., Mitchell E.P., Forsyth V.T., Gazit E., Seliktar D. (2012). The rheological and structural properties of Fmoc-peptide-based hydrogels: The effect of aromatic molecular architecture on self-assembly and physical characteristics. Langmuir.

[35] Chronopoulou L., Margheritelli S., Toumia Y., Paradossi G., Bordi F., Sennato S., Palocci C. (2015). Biosynthesis and characterization of cross-linked Fmoc peptide-based hydrogels for drug delivery applications. Gels.

[36] Chin D.-H., Woody R.W., Rohl C.A., Baldwin R.L. (2002). Circular dichroism spectra of short, fixed-nucleus alanine helices. Proc. Natl. Acad. Sci. USA.

[37] Sahoo J.K., Roy S., Javid N., Duncan K., Aitken L., Ulijn R.V. (2017). Pathway-dependent gold nanoparticle formation by biocatalytic self-assembly. Nanoscale.

[38] Ryan K., Beirne J., Redmond G., Kilpatrick J.I., Guyonnet J., Buchete N.-V., Kholkin A.L., Rodriguez B.J. (2015). Nanoscale piezoelectric properties of self-assembled Fmoc–FF peptide fibrous networks. ACS Appl. Mater. Interfaces.

[39] Goormaghtigh E., Ruysschaert J.-M., Raussens V. (2006). Evaluation of the information content in infrared spectra for protein secondary structure determination. Biophys. J..

[40] Zandomeneghi G., Krebs M.R.H., McCammon M.G., Fändrich M. (2004). FTIR reveals structural differences between native β-sheet proteins and amyloid fibrils. Protein Sci..

[41] Arrondo J.L.R., Goñi F.M. (1999). Structure and dynamics of membrane proteins as studied by infrared spectroscopy. Prog. Biophys. Mol. Biol..

[42] Sardaru M.-C., Marangoci N.-L., Palumbo R., Roviello G.N., Rotaru A. (2023). Nucleic acid probes in bio-imaging and diagnostics: Recent advances in ODN-based fluorescent and surface-enhanced Raman scattering nanoparticle and nanostructured systems. Molecules.

[43] Lakshmanan A., Cheong D.W., Accardo A., Di Fabrizio E., Riekel C., Hauser C.A.E. (2013). Aliphatic peptides show similar self-assembly to amyloid core sequences, challenging the importance of aromatic interactions in amyloidosis. Proc. Natl. Acad. Sci. USA.

[44] McFetridge M.L., Kulkarni K., Hilsenstein V., Del Borgo M.P., Aguilar M.-I., Ricardo S.D. (2023). A comparison of fixation methods for SEM analysis of self-assembling peptide hydrogel nanoarchitecture. Nanoscale.

[45] Falcone N., Ermis M., Tamay D.G., Mecwan M., Monirizad M., Mathes T.G., Khademhosseini A. (2023). Peptide hydrogels as immunomaterials and their use in cancer immunotherapy delivery. Adv. Healthc. Mater..

[46] He R., Li M., Li W., Li W., Xiao S., Cao Q., Luo S. (2025). Sustained release of αO-conotoxin GeXIVA [1,2] via hydrogel microneedle patch for chronic neuropathic pain management. Mar. Drugs.

[47] Coin I., Beyermann M., Bienert M. (2007). Solid-phase peptide synthesis: From standard procedures to the synthesis of difficult sequences. Nat. Protoc..

[48] Sunde M., Serpell L.C., Bartlam M., Fraser P.E., Pepys M.B., Blake C.C.F. (1997). Common core structure of amyloid fibrils by synchrotron X-ray diffraction. J. Mol. Biol..

[49] Jorbágy A., Király K. (1966). Chemical characterization of fluorescein isothiocyanate-protein conjugates. Biochim. Biophys. Acta Gen. Subj..

