# Supplementary material for: Investigating the Interactions of Peptide Nucleic Acids with Multicomponent Peptide Hydrogels for the Advancement of Healthcare Technologies
Source: Gels. 2025 May 17;11(5):367. doi: 10.3390/gels11050367 (PMC12111274; doi:10.3390/gels11050367)
Supplement: Supplementary file 1 [file gels-11-00367-s001.zip › gels-3592339-supplementary.pdf]

Supplementary Materials

Investigating the Interactions of Peptide Nucleic Acids with Multicomponent Peptide Hydrogels for the Advancement of Healthcare Technologies

Sabrina Giordano [1], Monica Terracciano [2,*], Enrico Gallo [1], Carlo Diaferia [2], Andrea Patrizia Falanga [2], Antonella Accardo [2], Monica Franzese [1], Marco Salvatore [1], Gennaro Piccialli [2], Nicola Borbone [2] and Giorgia Oliviero [3,*]

1 IRCCS SYNLAB SDN, Via G. Ferraris 144, 80143 Naples, Italy

2 Department of Pharmacy, University of Naples Federico II, Via D. Montesano 49, 80131 Naples, Italy

3 Department of Molecular Medicine and Medical Biotechnologies, University of Naples Federico II, Via S. Pansini 5, 80131 Naples, Italy

* Correspondence: monica.terracciano@unina.it (M.T.); giorgia.oliviero@unina.it (G.O.).

A)

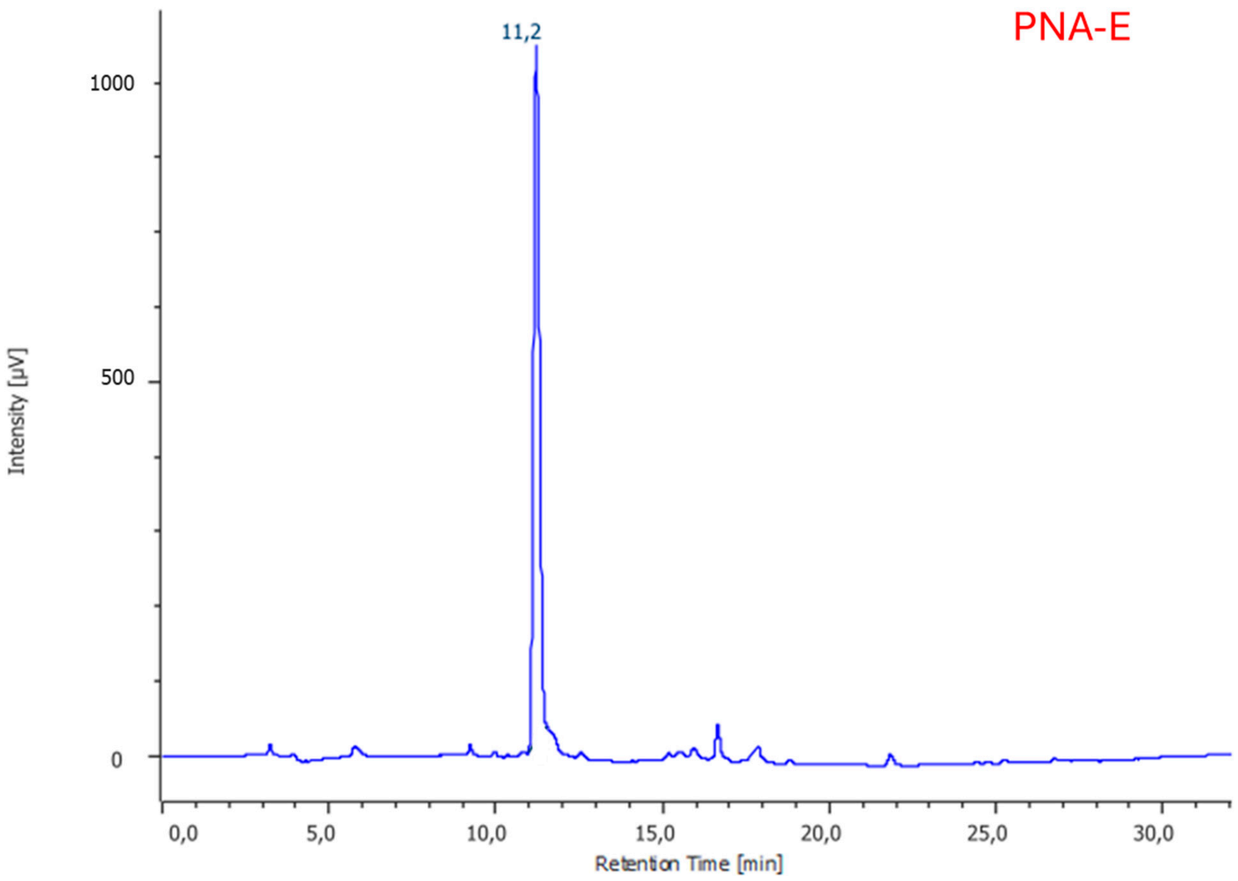

B)

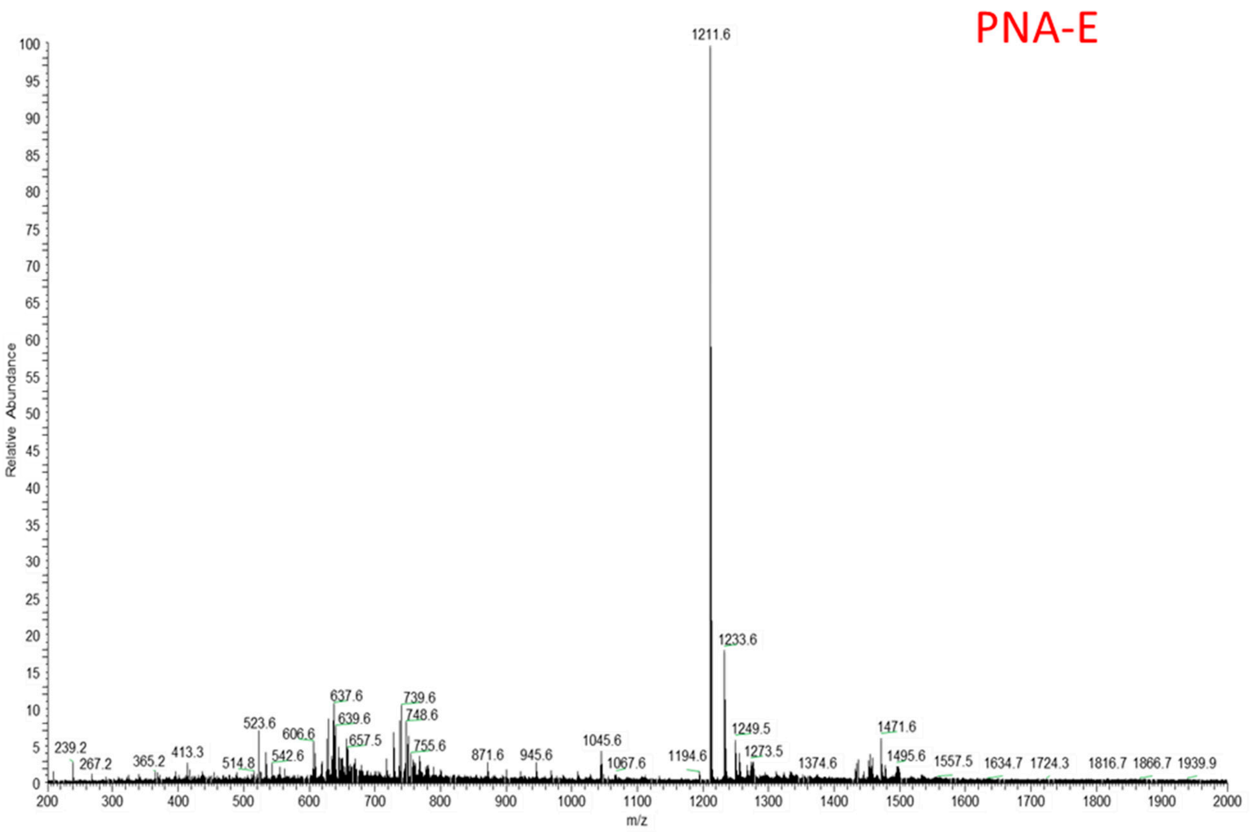

Figure S1. Physicochemical characterization of PNA-E: **(A)** RP-HPLC chromatography and **(B)** ESI mass spectrum.

A)

B)

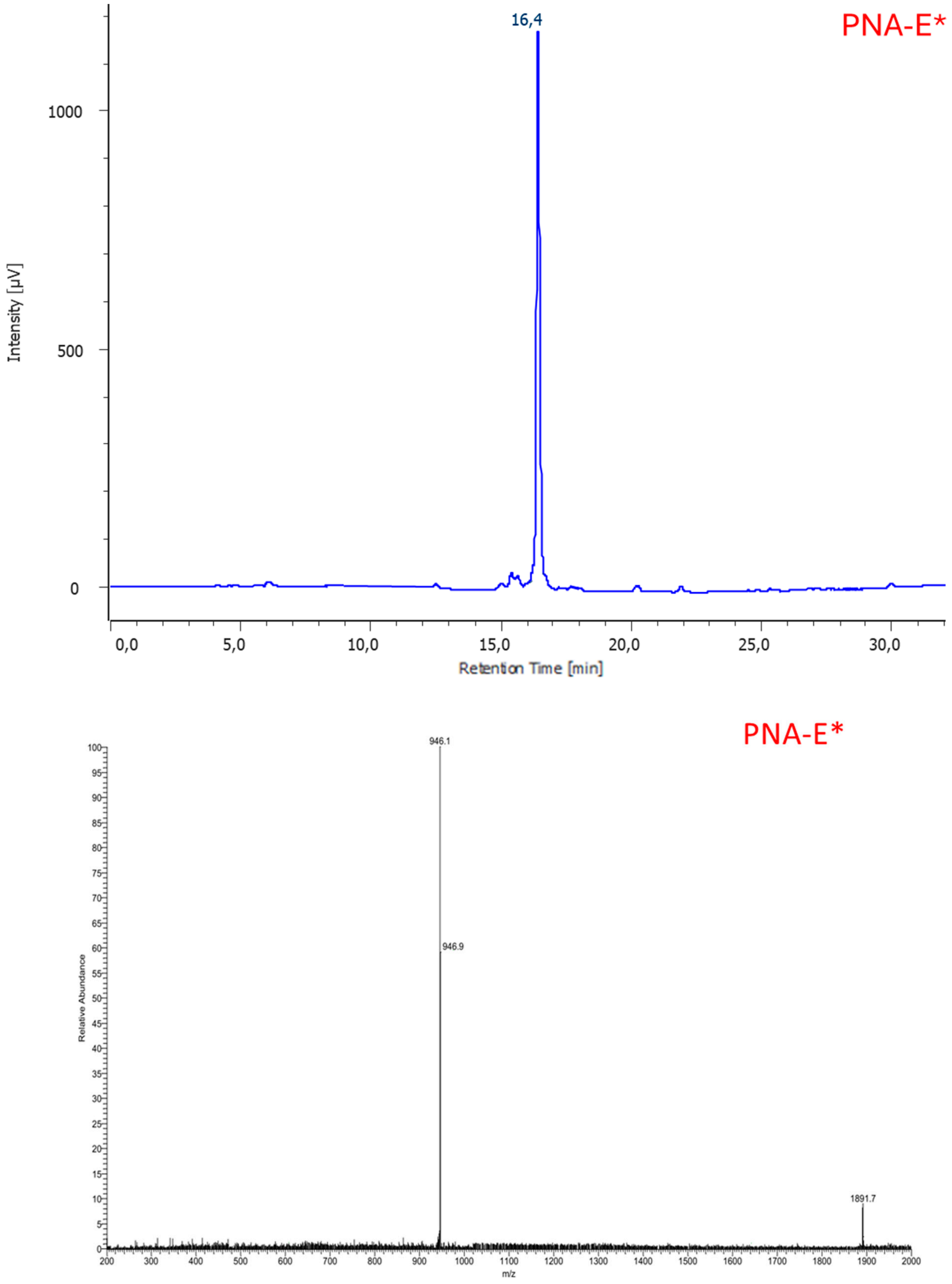

Figure S2. Physicochemical characterization of PNA-E*: **(A)** RP-HPLC chromatography and **(B)** ESI mass spectrum.

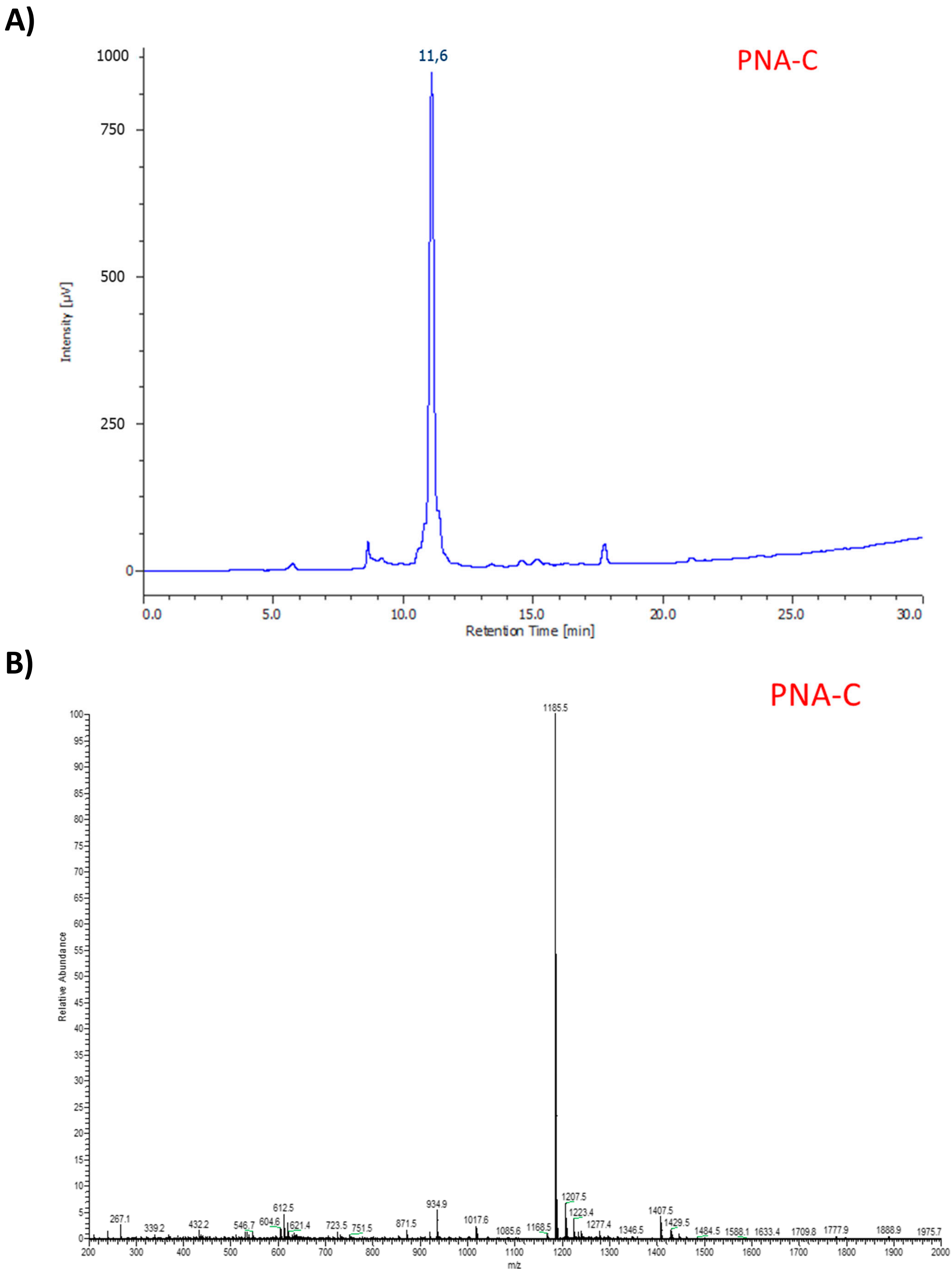

Figure S3. Physicochemical characterization of PNA-C: **(A)** RP-HPLC chromatography and **(B)** ESI mass spectrum.

A)

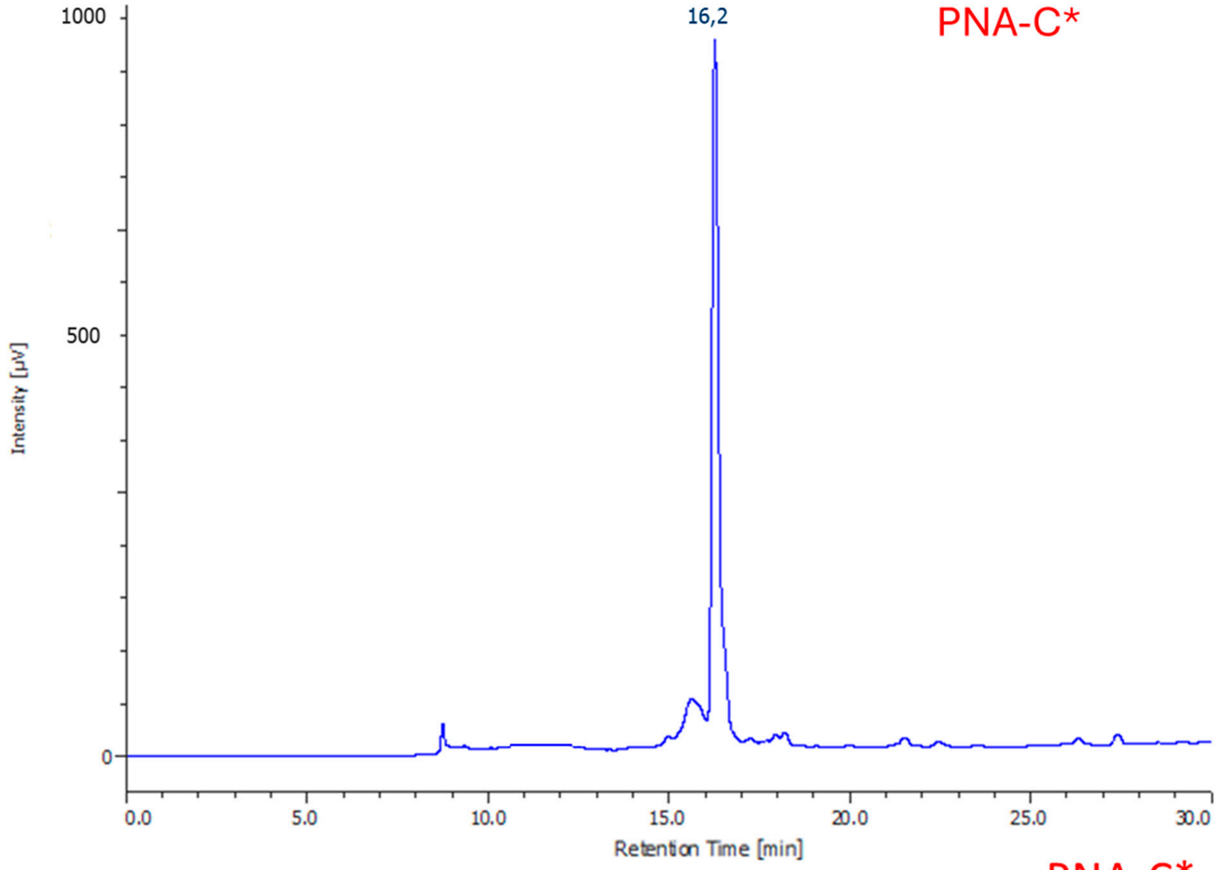

B)

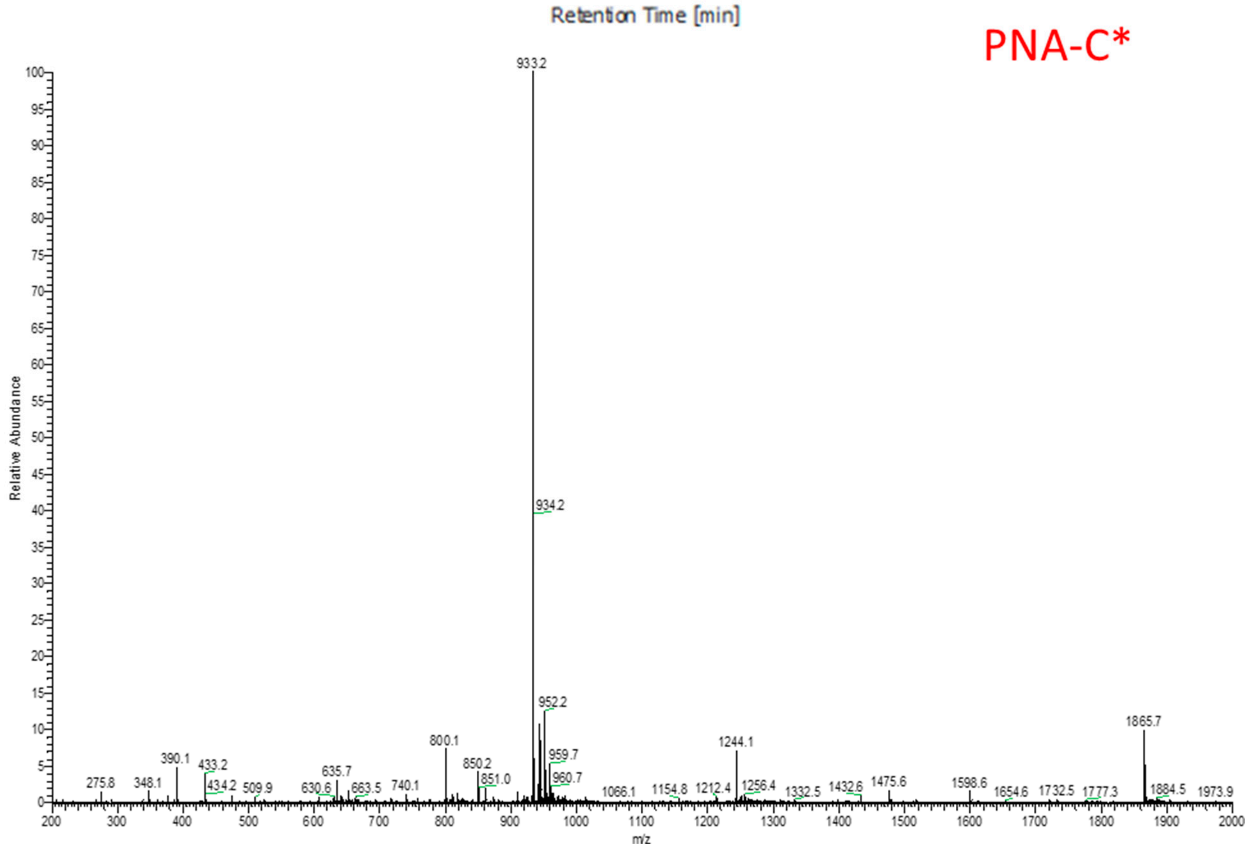

Figure S4. Physicochemical characterization of PNA-C*: **(A)** RP-HPLC chromatography and **(B)** ESI mass spectrum.

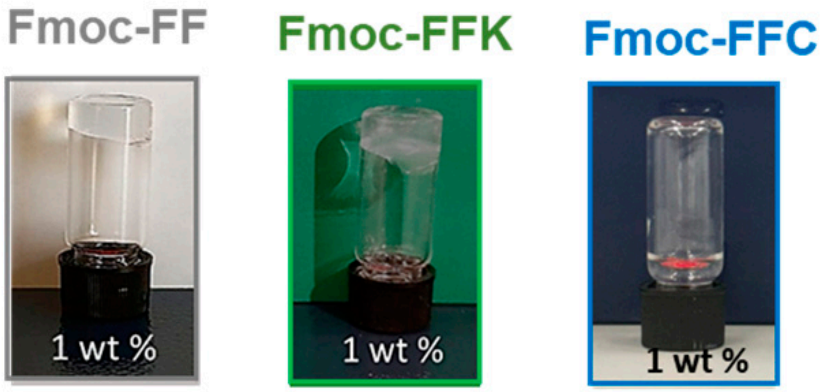

Figure S5. Inverted test tubes of each peptide alone (Fmoc-FF, Fmoc-FFK and Fmoc-FFC) at 1.0 wt%.

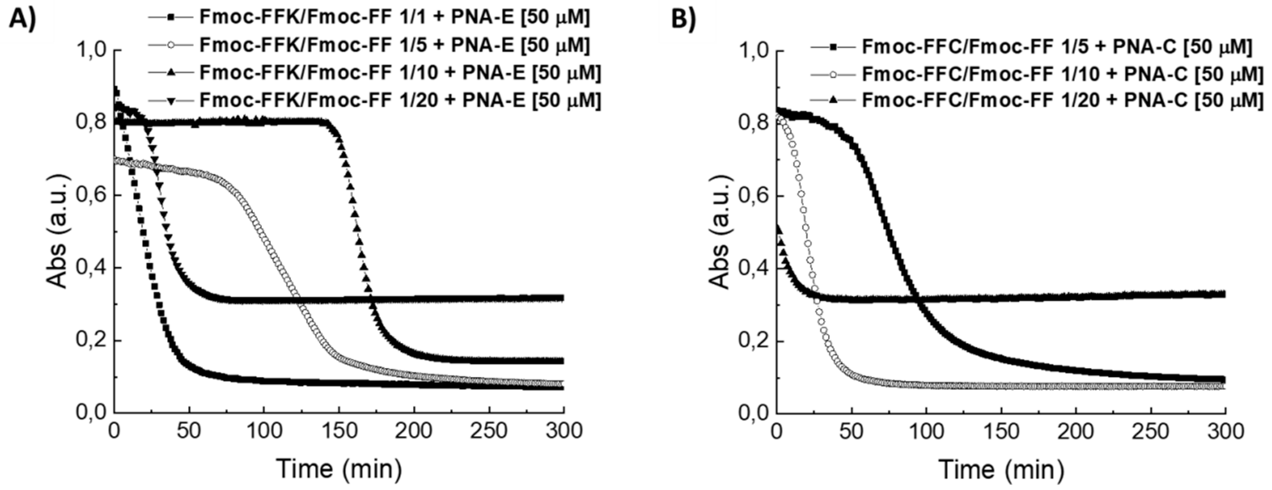

Figure S6. UV-*Vis* profiles of hydrogels obtained by mixing Fmoc-FFK **(A)** or Fmoc-FFC (**B**) with increasing amounts of Fmoc-FF (1/1, 1/5, 1/10, and 1/20 *w/w*) followed by addition of 50 μM PNA-E or PNA-C, respectively, in the long range (600 nm) as a function of time.

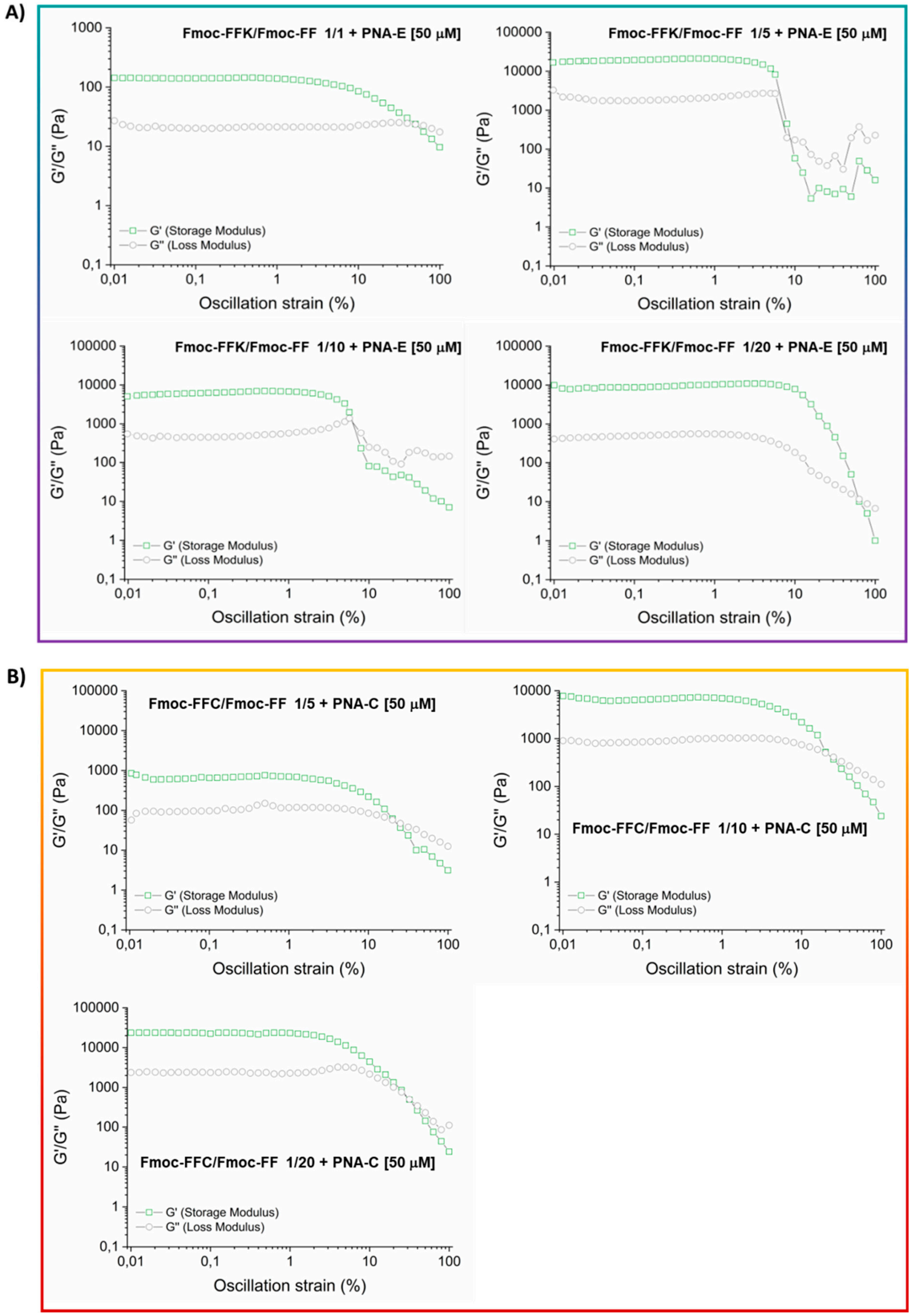

Figure S7. Time sweep (20 minutes) for: **(A)** PNA-E and **(B)** PNA-C [50μM] into mixed K or C hydrogels, respectively, (at different *w/w* ratios with Fmoc-FF: 1/1, 1/5, 1/10, 1/20). Rheological analysis is reported in terms of *G'* (Storage modulus) and *G''* (Loss modulus).

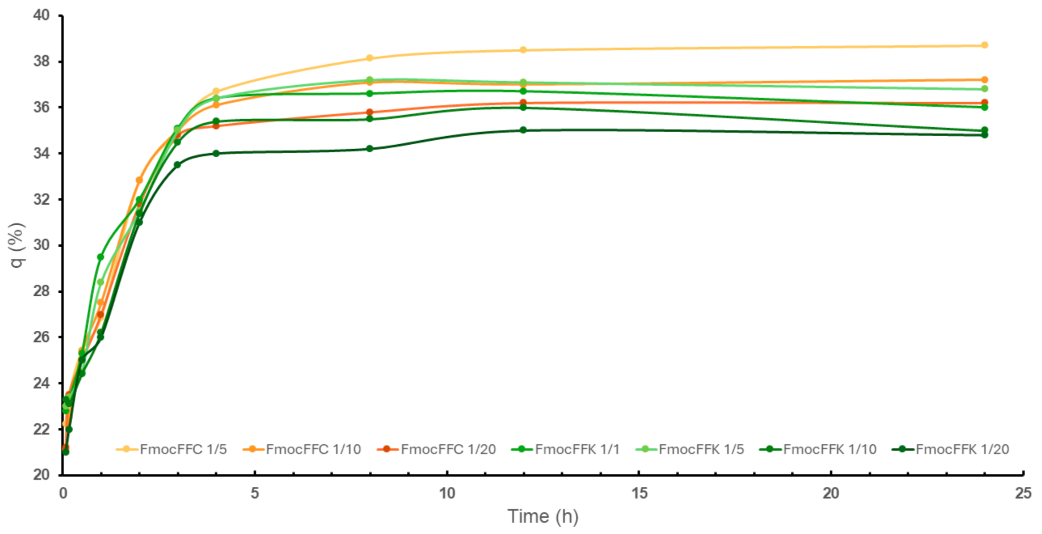

Figure S8. Swelling kinetics for Fmoc-FFK/Fmoc-FF and Fmoc-FFC/Fmoc-FF matrices. In the legend the Fmoc-FFX component is reported.

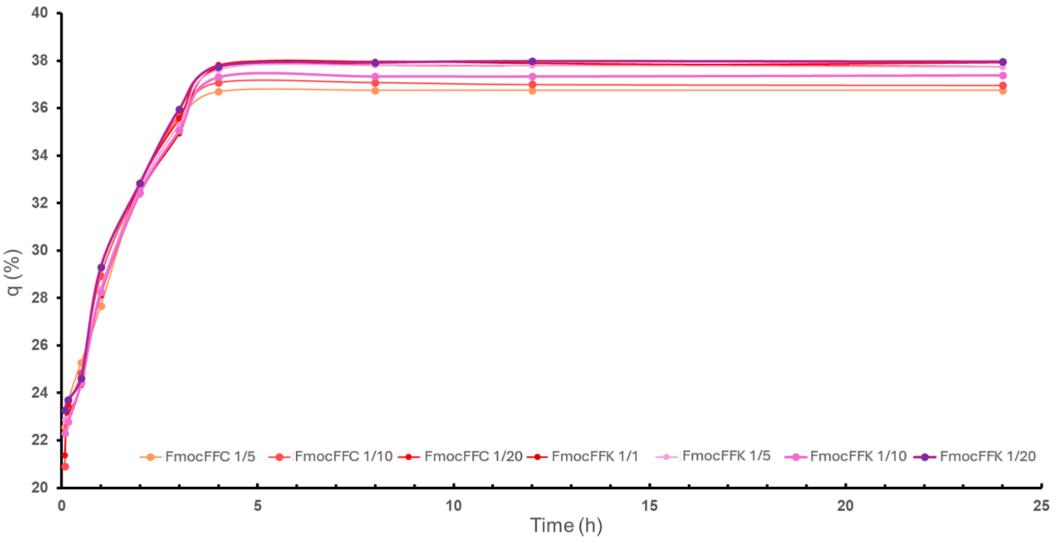

Figure S9. Swelling kinetics for Fmoc-FFK/Fmoc-FF and Fmoc-FFC/Fmoc-FF matrices, containing PNA-C and PNA-E [50 µM], respectively. The legend only shows the Fmoc-FFX component.

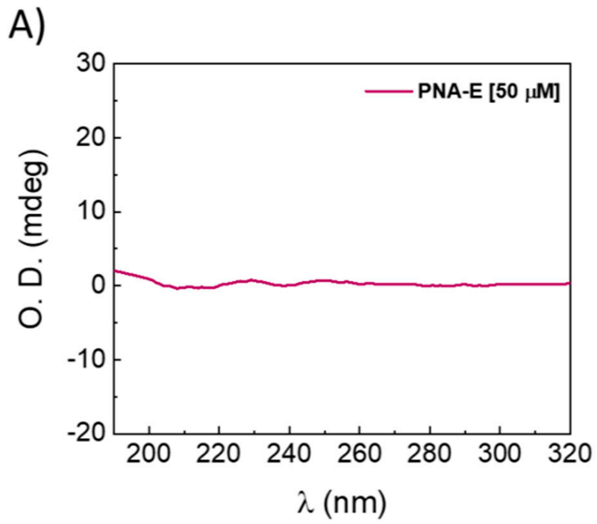

Figure S10. CD spectra (range 320-190 nm) **(A)** of PNA-E [50 µM] and **(B)** of PNA-C [50 µM].

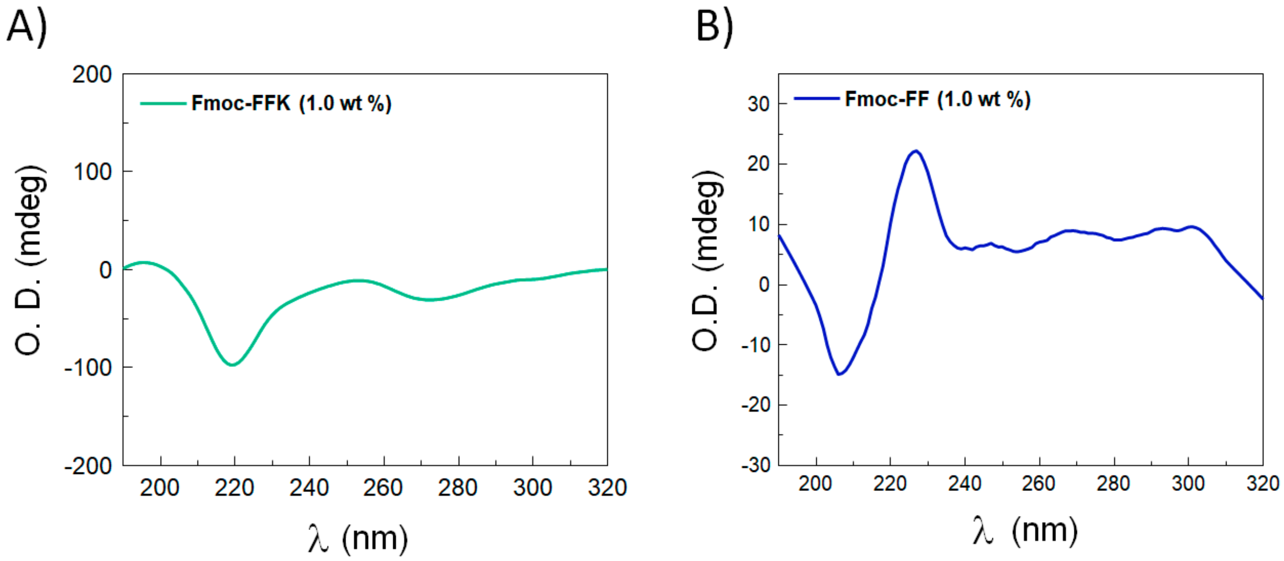

Figure S11. CD spectra (range 320-190 nm) of **(A)** Fmoc-FF and **(B)** Fmoc-FFK at 1.0 wt%.

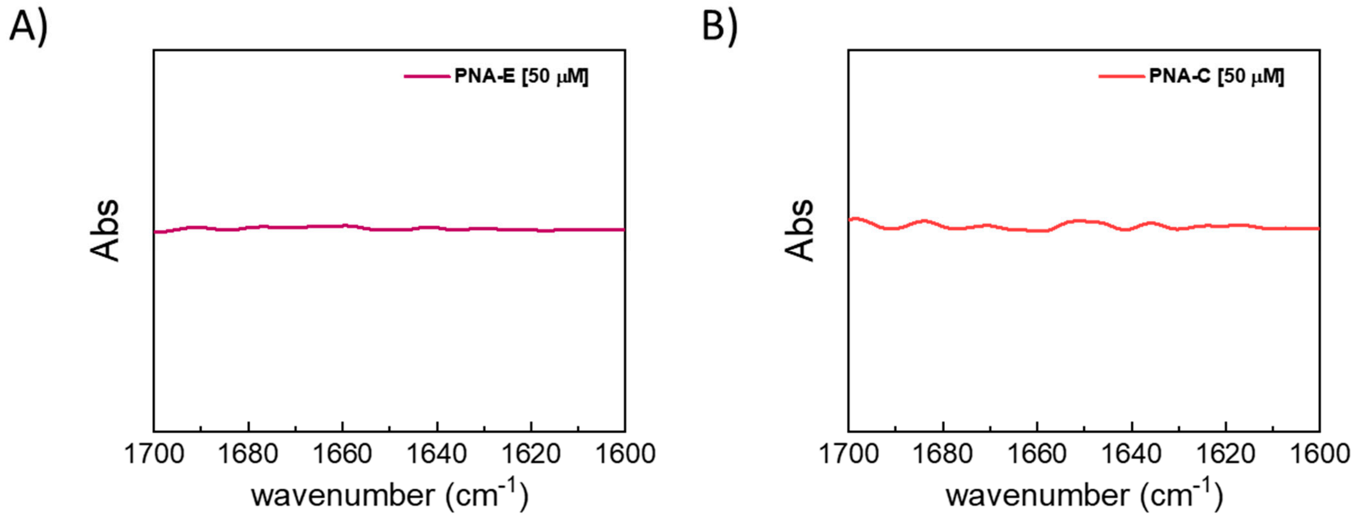

Figure S12. FT-IR absorbance spectra in the Amide I region (range 1700–1600 cm^{-1}) of (A) PNA-E [50 μM] and (B) PNA-C [50 μM].

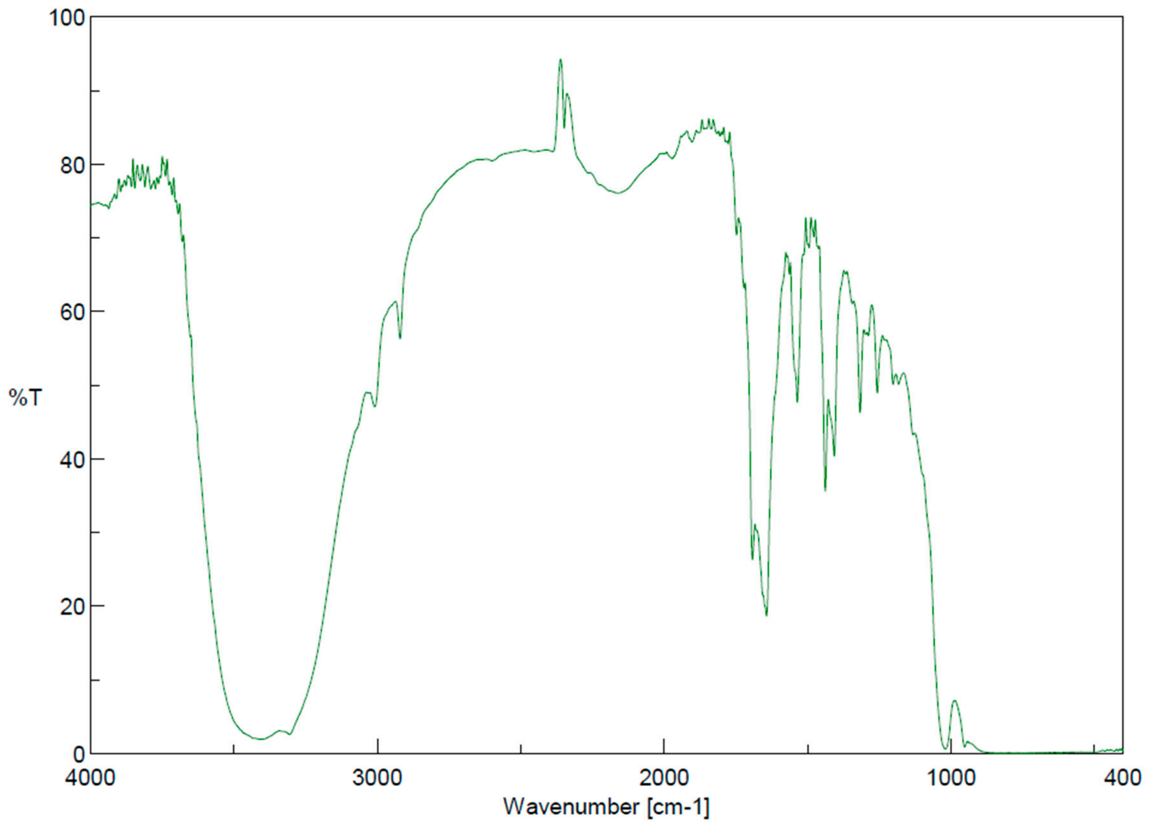

Figure S13. FTIR spectrum collected over the full mid-infrared range (4000–400 cm^{-1}) of Fmoc-FFK/Fmoc-FF 1/1 + PNA-E [50 μM].

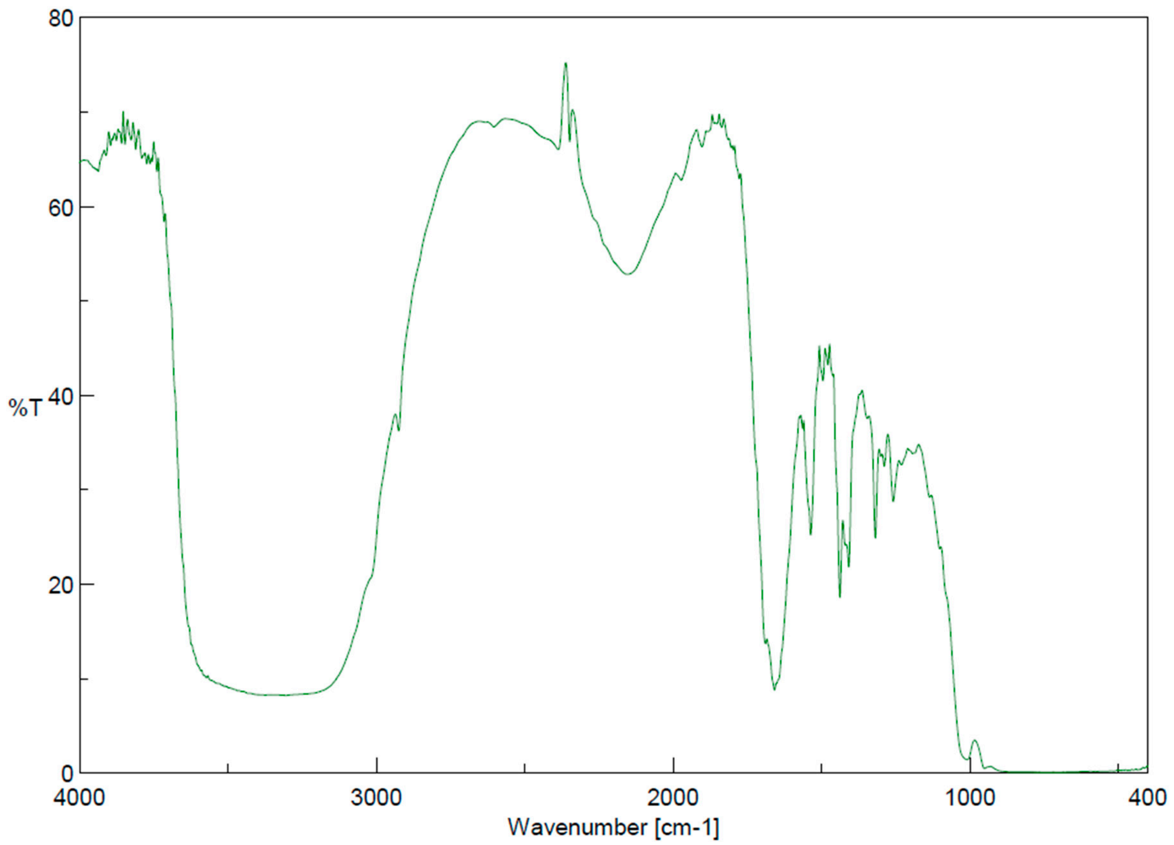

Figure S14. FTIR spectrum collected over the full mid-infrared range (4000–400 cm^{-1}) of Fmoc-FFK/Fmoc-FF 1/20 + PNA-E [50 μM].

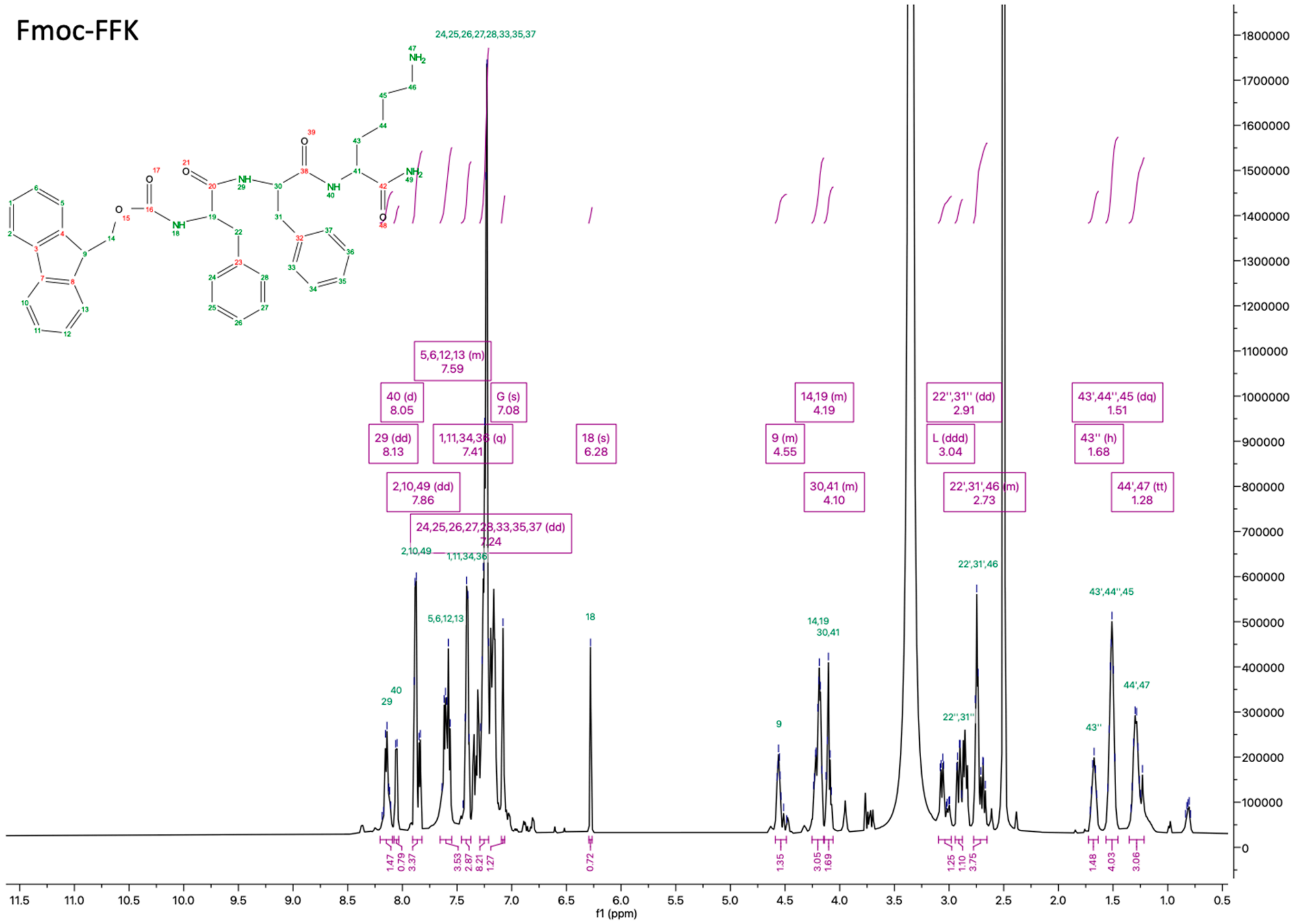

Figure S15. ^{1}H-NMR spectrum of Fmoc-FFK in DMSO-d$_6$.

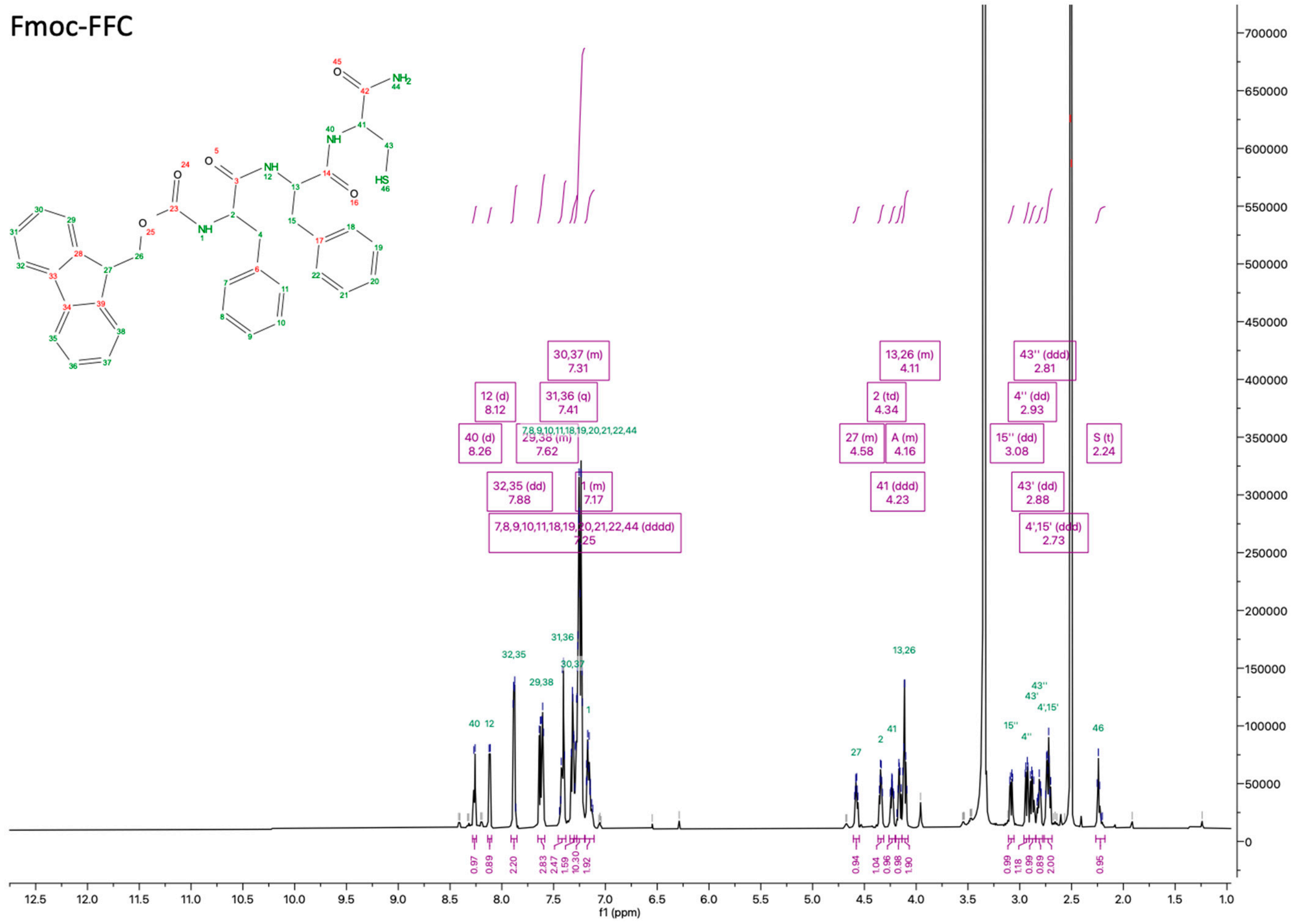

Figure S16. ^{1}H-NMR spectrum of Fmoc-FFC in DMSO-d$_6$.

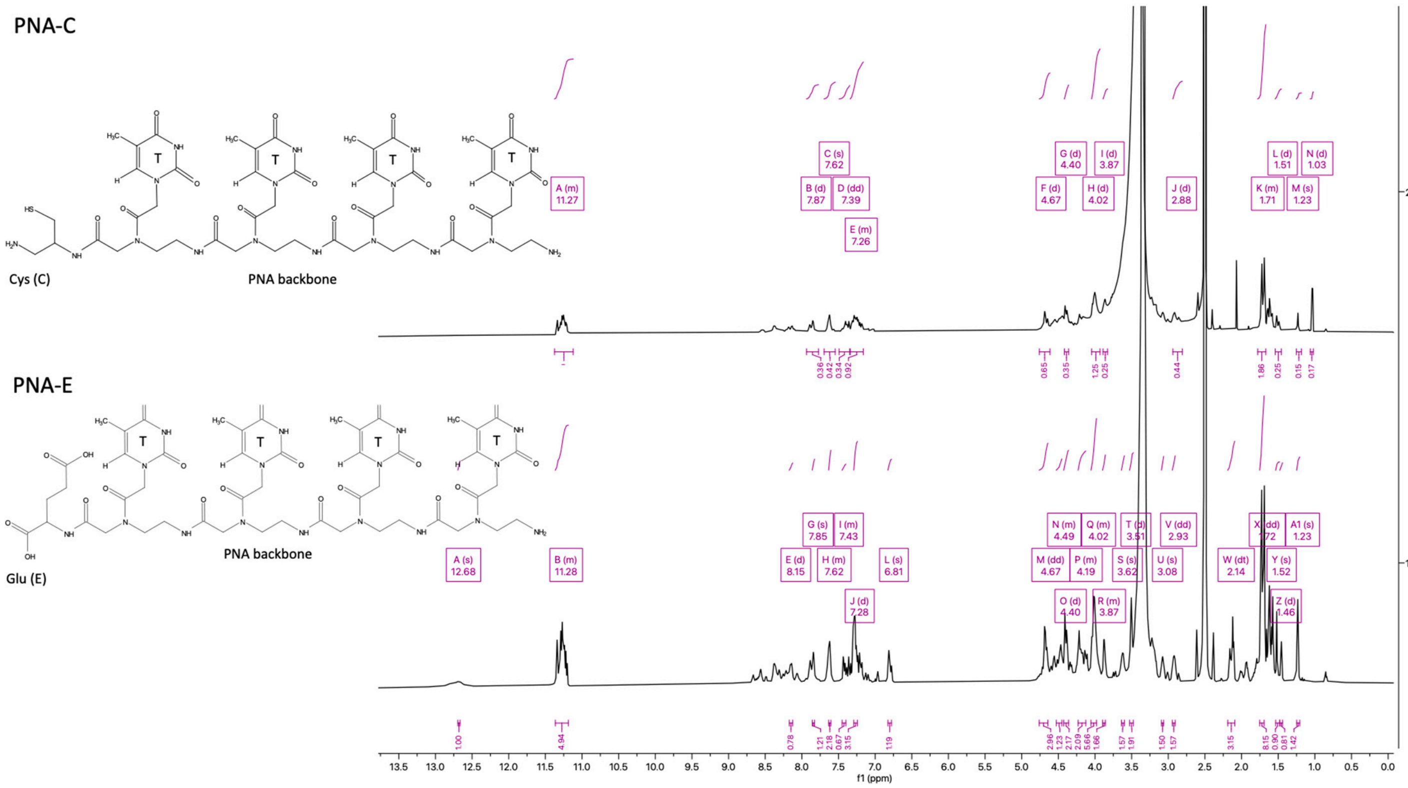

Figure S17. ^{1}H-NMR spectrum of PNA-C and PNA-E in DMSO-d_6 with chemical structure, respectively.

Table S1 of ^{1}H NMR Chemical Shifts for PNA-C and PNA-E in DMSO-d_6

Proton	Chemical Shift (ppm)
H6 (pyrimidine - thymine)	7.5 - 7.8
NH (amide, PNA backbone)	8.5 - 10.0
CH_2 (methylene, PNA backbone)	3.0 - 4.5
CH_3 (methyl group of thymine)	1.6 - 1.9
COOH (Glu)	~12.0
SH (Cys)	~1.3 - 1.5

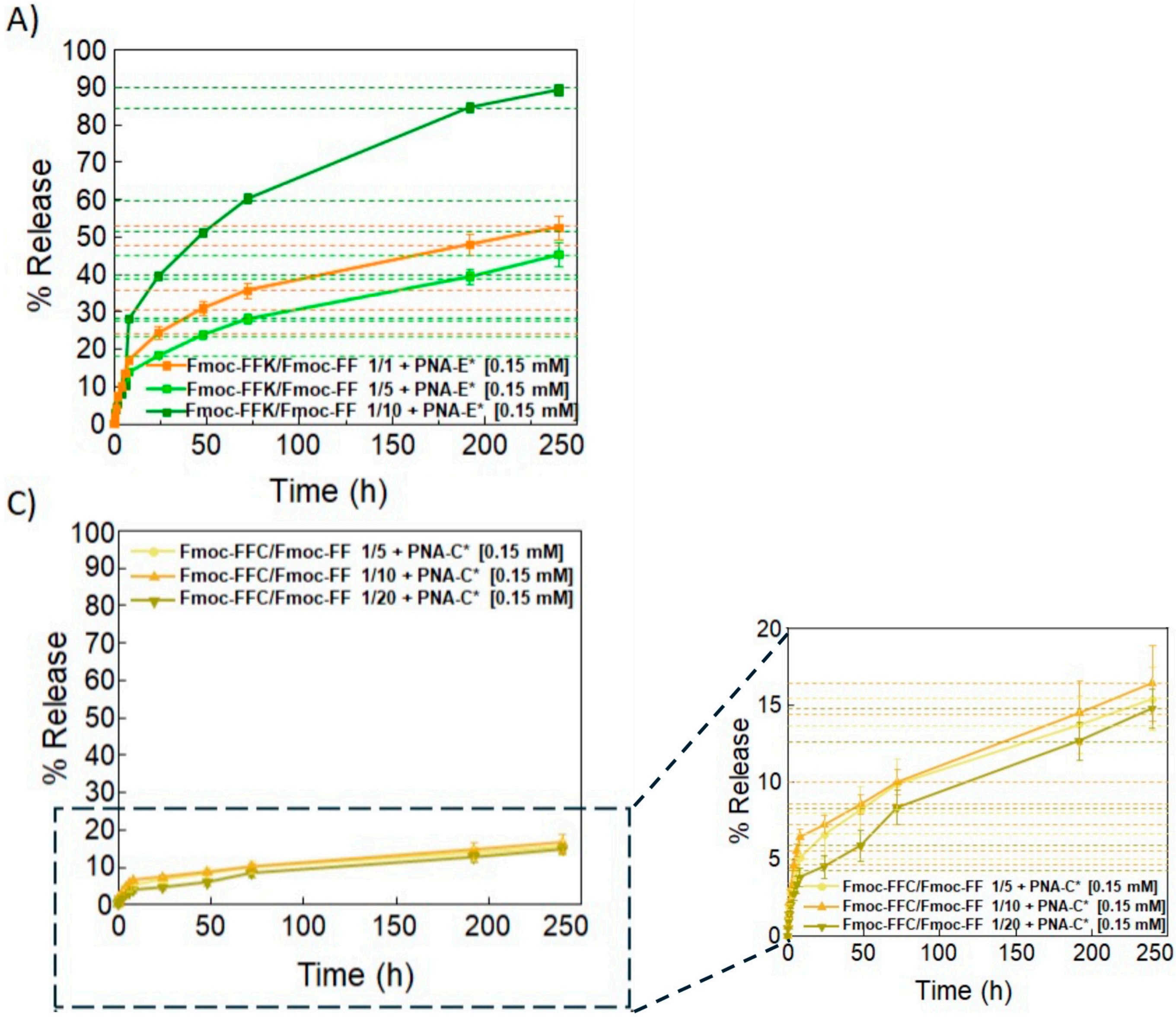

Figure S18. Release kinetics of PNA-E* and PNA-C* (0.15 mM) from peptide-based hydrogels over a 10-day period are shown in panels A and C, respectively. Dashed lines parallel to the x-axis are included to support qualitative assessment of the slope, highlighting differences in release profiles associated with electrostatic (PNA-E*) and covalent (PNA-C*) interactions.

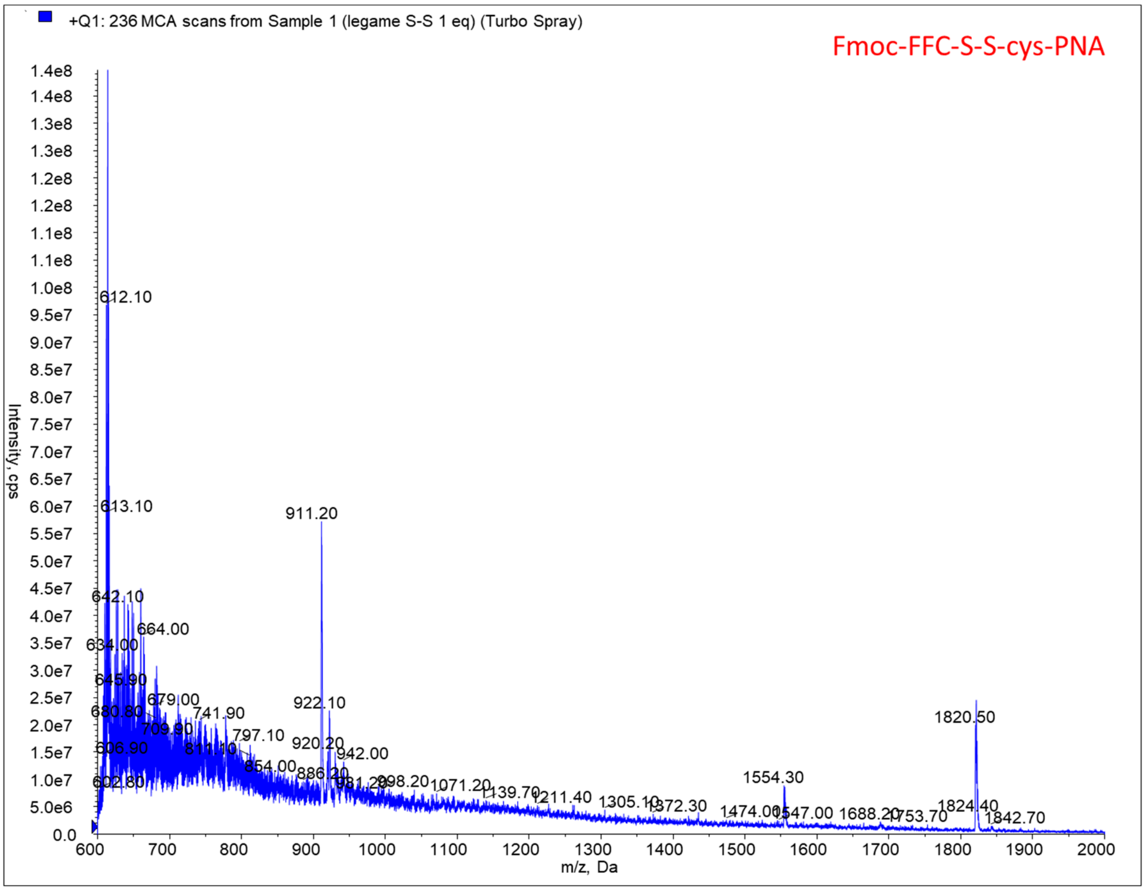

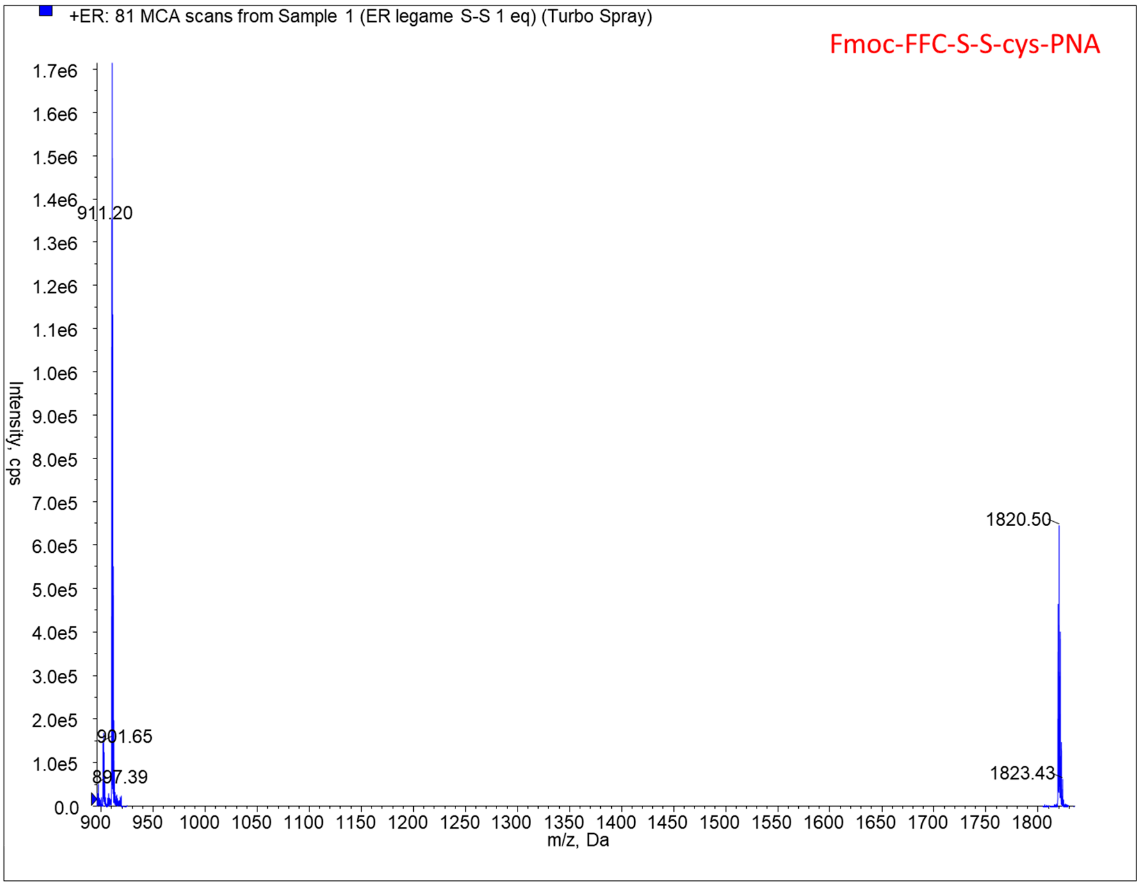

Figure S19. Physicochemical characterization of Fmoc-FFC-S-S-cys-PNA (1.0 EQ) *via* ESI mass spectrometry (Enhanced Resolution).
